# Mechanical Behavior of Grouted Fractured Sandy Mudstone Under Different Grouting Pressures: Experimental Investigation and CT-Based In Situ Numerical Modeling

**DOI:** 10.3390/ma19050840

**Published:** 2026-02-24

**Authors:** Yuxu Shen, Zhaoyun Chai, Xu Liu, Chang Xiao, Tianyu Li, Xiangyu Liu, Junqing Guo

**Affiliations:** 1 Key Laboratory of In-Situ Property-Improving Mining of Ministry of Education, Taiyuan University of Technology, Taiyuan 030024, China; shenyx@sxie.edu.cn (Y.S.); 18348285346@163.com (X.L.); tyxchang@163.com (C.X.); hamlets___7@163.com (T.L.); 13753106501@163.com (J.G.); 2 Mining Engineering Department, Shanxi Institute of Energy, Jinzhong 030600, China; lxy2773@163.com

**Keywords:** fractured sandy mudstone, grouting pressure, CT scan, in situ modeling, mechanical properties

## Abstract

**Highlights:**

A numerical simulation methodology integrating “CT scanning—in situ modeling—mechanical analysis” was established, enabling in situ modeling of grout–rock composite structures.

The nonlinear enhancement law of grouting pressure on the mechanical properties of fractured sandy mudstone was revealed, and 3 MPa was determined as the optimal grouting pressure.

The transition of the rock failure mode from brittle to ductile behavior induced by grouting was elucidated.

**Abstract:**

To investigate the effect of different grouting pressures on the reinforcement of fractured sandy mudstone, grouting tests, mechanical experiments, CT scanning, and SEM analysis were conducted on fractured rock samples. Based on CT data, the precise internal structure of the grouted rock samples was obtained. High-fidelity numerical models were constructed in ABAQUS through image processing and mesh mapping techniques and then imported into ANSYS for uniaxial compression simulation. The results showed that under grouting pressures of 1 MPa, 3 MPa, and 5 MPa, the compressive strengths of the samples were 10.08 MPa, 12.39 MPa, and 13.28 MPa, corresponding to increases of 22.9% and 7.2%, respectively. The elastic moduli were 1.16 GPa, 1.52 GPa, and 1.63 GPa, with increases of 31% and 7.2%, respectively. The toughness index and brittleness index exhibited opposite trends: the toughness index increased from 1.6555 to 1.7135 and then to 1.7648 (rises of 3.5% and 2.9%), while the brittleness index decreased from 1.5255 to 1.4020 and then to 1.3075 (reductions of 8.1% and 6.7%). The ductility index rose from 1.8760 to 2.0972 and then to 2.2637 (increases of 11.8% and 7.9%). The failure mode of the grouted rock samples shifted from brittle to ductile behavior, with the most pronounced overall mechanical improvement observed at 3 MPa grouting pressure. SEM analysis indicated that as the grouting pressure increased, the dominant crack type changed from large cracks to micro-cracks. At 3 MPa, the grout fully penetrated micro-pores and enhanced the sample’s integrity, whereas at 5 MPa, excessive grouting pressure induced damage to the rock matrix itself. Fracture simulations further demonstrated that as the grouting pressure increased from 1 MPa to 3 MPa and above, the failure mode shifted from being controlled by pre-existing fractures to a holistic rupture involving both the grout and the rock matrix, leading to significantly improved structural integrity. This study establishes an integrated numerical simulation approach of “CT scanning—in situ modeling—mechanical analysis”, providing a scientific basis for optimizing grouting parameters.

## 1. Introduction

Sandy mudstone, a typical engineering surrounding rock, is widely distributed in mines, tunnels, slopes, and other projects in China. However, the reinforcement of fractured rock masses presents a persistent core challenge in the fields of rock mechanics and geotechnical engineering. These rock masses often contain inherent defects such as joints and fractures, which significantly compromise their overall strength and stability, posing serious threats to underground excavations, slope stability, and mining safety [1,2,3]. Grouting technology is an effective method for reinforcing such fractured rock masses. The grout enhances the integrity and mechanical properties of the rock by filling the fractures [4,5,6]. Grouting pressure is a critical factor influencing the reinforcement efficacy. Insufficient pressure hinders effective grout diffusion and penetration, while excessive pressure may cause rock fracturing, escalate costs, or even induce new damage. Therefore, systematically investigating the reinforcement effects of different grouting pressures on fractured sandy mudstone and elucidating the underlying mechanical mechanisms hold significant theoretical value and provide important guidance for engineering practice [7,8,9].

Regarding research on the mechanical properties of grouted reinforcement and CT scanning, Jianhua Fu et al. studied the effectiveness of fracture grouting technology for compacting deep coal seams through triaxial compression tests and industrial CT scanning [10]. Xin Wang et al. utilized a self-developed true triaxial grouting device to conduct grouting under full-stress states on freeze–thaw fractured rocks and performed CT-based permeability simulations on the grouted rock mass [11]. Xiangrui Qin et al. revealed the influence of grouting materials on the meso-structure and macroscopic mechanical behavior of the grout–rock interface through a series of experiments, employing Nuclear Magnetic Resonance (NMR) and CT scanning to study the pore composition and meso-structure of grouted rock [12]. Changxing Zhu et al. determined the optimal carbon fiber (CF) content by conducting uniaxial compression tests on rock samples containing 0%, 0.5%, 1.0%, and 1.5% CF and established correlations between micro-mechanics and macro-mechanics using CT and PFC2D simulation analyses [13]. Ji-Peng Wang et al. investigated the performance of microbially induced calcium carbonate precipitation (MICP) under different grouting saturation conditions. CT test results indicated that the contact surface area became more uniform and the coordination number increased after cementation [14]. Xin Wang et al., aiming to explore the grout diffusion mechanisms of different materials in freeze–thaw rock masses, conducted true triaxial stress field grouting experiments on freeze–thaw fractured rock. They performed CT scanning and Scanning Electron Microscopy (SEM) on grouted specimens to obtain 3D reconstructions of grout diffusion patterns and vein networks under different freeze–thaw conditions [15].

In the field of numerical simulation for grouting reinforcement, Shiqi Liu et al. conducted experimental and numerical studies using high-water-content grouting materials, based on the working principles of permeation grouting and grouting pipe technology [16]. Zhenhua Jiang et al., combining parameters from field grouting tests, performed multi-factor numerical simulations to clarify the evolution laws of surrounding rock stress and displacement under different parameters [17]. I. Sakhno et al., considering the influence of moisture content on rock properties, used numerical simulation methods to study the stress and strain distribution in surrounding rock, taking floor heave as a case study combined with grouting control technology [18]. M. Motamedi et al. determined the optimal size of mine pillars in the Faryab mine through numerical simulation and utilized grouting technology to reduce pillar dimensions [19]. Hui Wang et al. established a finite element model in ABAQUS for the original support scheme and determined the rheological parameters of surrounding rock using a back-analysis method based on field-monitored deformation data [20]. Changxiang Wang et al. identified the stability of overlying strata and the grouted body as two key factors affecting the stability of grouted goafs under building loads and verified the stability of the grouting reinforcement project through numerical simulation [21].

While these studies have yielded valuable insights, they often fall short of accurately reflecting the heterogeneous distribution of grout within complex fracture networks and its controlling effect on the mechanical behavior of the rock mass [22,23,24]. With the advancement of non-destructive testing technologies like CT, it has become possible to acquire the detailed 3D internal structure of materials without causing damage. By integrating CT data with numerical simulation software such as ABAQUS (2021) and ANSYS (2022R1), it is feasible to construct in situ models that realistically represent the geometry of fractures and material interfaces. This “in situ modeling” approach preserves the structural information of the rock mass to the greatest extent, offering a novel pathway for investigating rock failure behavior [25,26,27]. However, the existing literature lacks the application of this methodology to study the grouting reinforcement effectiveness and corresponding mechanical response mechanisms specifically in fractured sandy mudstone. This paper, based on the mechanical properties, CT scan data, and SEM micro-morphology of grouted fractured sandy mudstone, constructs a finite element model in ABAQUS that reflects the authentic internal structure. This model is subsequently analyzed in ANSYS for simulating mechanical behavior. By analyzing the failure process, crack evolution patterns, stress contours, and other aspects, this study systematically investigates the enhancement effects of different grouted pressures on the macroscopic mechanical properties of fractured sandy mudstone. It reveals the crack evolution mechanisms during the fracturing of grouted rock samples and identifies the optimal grouting pressure. The findings of this research can provide a reference basis for the design and practice of grouting reinforcement in geotechnical engineering.

In summary, existing studies have limitations in accurately characterizing the heterogeneous distribution of grout within complex fracture networks and its controlling effect on the mechanical behavior of rock masses. This paper describes how, by integrating CT scanning, image processing, and numerical simulation (ABAQUS/ANSYS/LS-DYNA), an “in situ modeling” framework capable of realistically representing fracture geometry and material interfaces was constructed to overcome the aforementioned limitations. Furthermore, this study emphasizes that fractured sandy mudstone, as a typical engineering surrounding rock, is crucial for the safety of mining, tunneling, and other engineering projects. Its unique mineral composition and fracture characteristics make research into its response mechanisms to grouting reinforcement highly significant.

## 2. Materials and Methods

### 2.1. Rock Samples and Grouting Materials

**Figure 1:**
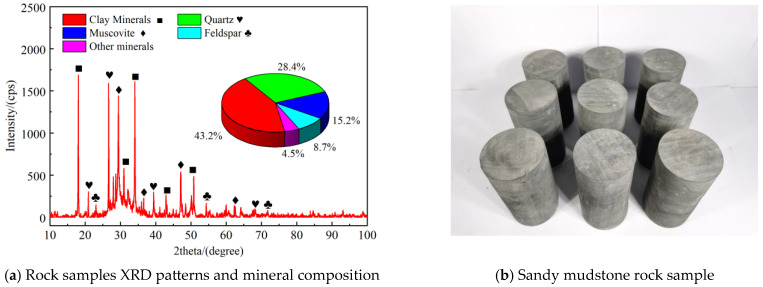
Rock samples and XRD analysis.

The rock was obtained from the roof of the No. 10 coal seam in the Baigou Coal Mine, Lingshi County, Jinzhong City, Shanxi Province, China. Thin-section identification revealed that the rock is a dark gray, laminated siltstone. X-ray diffraction (XRD) analysis indicated that the rock contains 43.2% clay minerals, 28.4% quartz, 15.2% muscovite, 8.7% feldspar, and 4.5% other minerals. Its diffraction pattern and mineral composition are shown in Figure 1a. To reduce the impact of structural and compositional differences on the experimental results, all rock samples were drilled from a rock block at the same location in the coal mine. In accordance with the suggestions of the International Society for Rock Mechanics (ISRM) testing guidelines, nine standard rock samples with a diameter of 50 mm, a height of 100 mm, and an end flatness of less than 0.02 mm were prepared for the tests. The rock samples are shown in Figure 1b.

The study employed a self-developed nano-silica sol-modified cementitious grout as the grouting material [28]. The chemical compositions of ordinary Portland cement (From Taiyuan, China), aluminate cement (From Taiyuan, China), and fly ash (From Taiyuan, China) are presented in Table 1, while the optimal material proportions are detailed in Table 2. This material synergistically modifies conventional cement grout by adding nano-silica sol and EVA (Ethylene Vinyl Acetate), constructing a fibrous network structure with significant toughening effects within the hardened grout body. Compared with traditional cement-based grouting materials, this composite material has the advantages of high early strength and strong fracture toughness. The physical and mechanical parameters of the grouting material are shown in Table 3.

### 2.2. Experimental Methodology

**Figure 2:**
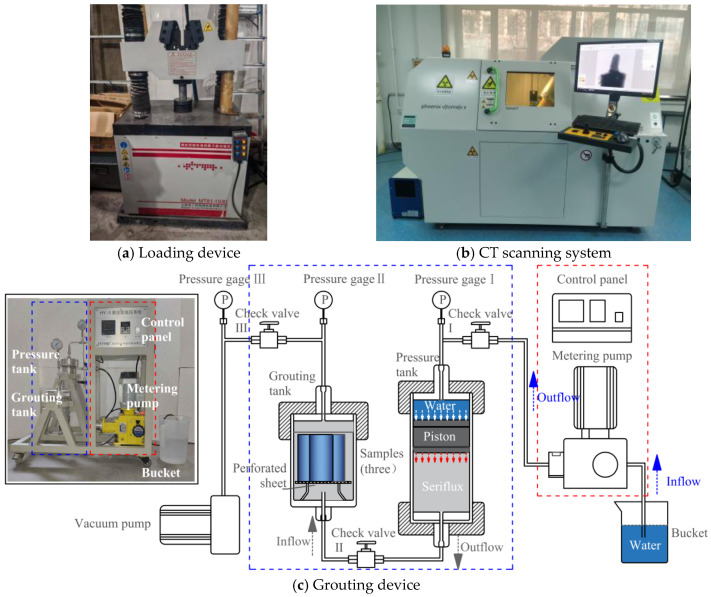
Experimental setup.

The test primarily consisted of a loading device, CT scanning (multiscale multiphysical-field coupled coal–rock scanning analysis system), and a grouting device, as shown in Figure 2. The loading device was an MT81-1030 type electro-hydraulic servo press, with a maximum axial load of 300 kN and a loading accuracy error of less than 1%. A uniaxial loading test was conducted on the rock samples using displacement control. The loading device is shown in Figure 2a, operating at a constant loading rate of 0.1 mm/s. Initial uniaxial compression tests were conducted on the nine specimens shown in Figure 1 to obtain the mechanical properties of the rock matrix. The uniaxial compressive strengths of the nine rock specimens were 44.85 MPa, 45.67 MPa, 43.56 MPa, 46.39 MPa, 45.22 MPa, 44.12 MPa, 46.71 MPa, 44.58 MPa, and 46.02 MPa, respectively. Subsequently, to ensure the repeatability of the tests and the reliability of the data, grouting reinforcement tests were performed sequentially on these specimens—which had undergone uniaxial compression testing (with prefabricated fractures)—under grouting pressures of 1 MPa, 3 MPa, and 5 MPa, with three tests conducted at each pressure level.

CT scanning was performed using a GE Phoenix V|tome|X S240 CT inspection system, equipped with a 180 kV/15 W high-power nanofocus X-ray source. The CT scanning device is shown in Figure 2b. The fractured rock sample was constrained and fixed on the CT sample stage using a heat shrink tube. It was positioned 250 mm from the X-ray tube and approximately 950 mm from the detector. The scanning voltage was set to 180 kV and the current to 150 μA, and a 360° high-precision scan was performed on it. The single-image exposure time was 1000 ms, with a resolution of approximately 50 μm. Avizo software was used to perform 3D reconstruction of the CT images to characterize the geometric morphology and spatial distribution characteristics of the internal fracture network in the rock sample.

The grouting equipment utilized a self-developed HY-II type pressure-boosting grouting testing machine. This apparatus mainly consisted of a metering pump, vacuum pump, pressure tank, grouting tank, pressure sensor, pressure gauge, control panel, and other components. The grouting device is shown in Figure 2c. During grouting, the fractured rock sample is first placed into the grouting tank and the top cover is tightened. The vacuum pump is then activated to evacuate the grouting tank to a vacuum state. Subsequently, the valve is opened, allowing the grout to flow from the pressure tank into the grouting tank through the pipeline under the pressure differential while the piston of the pressure tank simultaneously moves downward. Finally, the metering pump is activated. By pumping water into the upper chamber of the piston in the pressure tank, the piston is forced to move downward. This gradually increases the pressure inside the grouting tank to the set pressure value, achieving the pressure grouting operation. To ensure the pressure inside the grouting tank remained constant during the grouting process, the pressure sensor and control module automatically regulated the water inflow (0–300 mL/min) via a preset program, thereby maintaining the set pressure through automatic adjustment. The grouting pressures were 1 MPa, 3 MPa, and 5 MPa, respectively, with a grouting duration of 60 min. After grouting was completed, the pressure was released, and the grouted rock samples were placed in a constant temperature and humidity curing chamber (temperature: 20 °C, humidity: 95%) for 28 days of curing.

After the grouted rock mass was cured, a second CT scan was performed; finally, uniaxial loading tests were conducted again on the grouted rock samples to test and analyze their mechanical properties. The results were compared with those of the original rock samples to evaluate the effectiveness of the grouting reinforcement.

**Figure 3:**
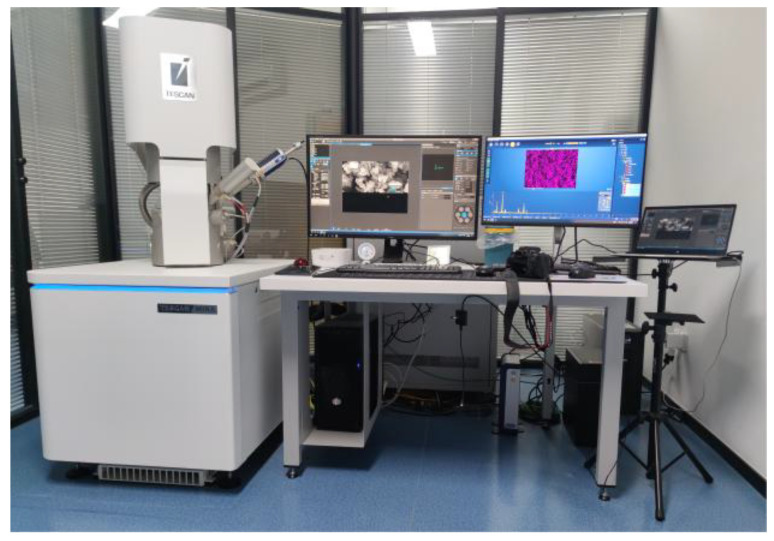
Scanning Electron Microscope (SEM) equipment.

After the grouted rock samples were fractured, three test blocks were taken along each failure surface, ensuring that each block had at least one face that was a contact surface between the grout and the rock. These were then processed into cubic samples with dimensions no larger than 10 mm × 10 mm × 10 mm. The samples were fixed on the specimen stage using conductive tape and sputter-coated with gold for 60 s. After the chamber was evacuated, their micromorphological features were imaged. This test utilized a Tescan Mira4 scanning electron microscope (SEM), with the equipment shown in Figure 3. The scanning voltage was 15 kV, using the SE detector for secondary electron imaging. Images were acquired at magnifications of 300×, 500×, 1000×, 2000×, and 5000×, respectively. After evacuation, microscopic morphology characteristics were photographed.

## 3. Analysis of Mechanical Properties of Rock Samples Before and After Grouting

### 3.1. Stress–Strain Curves of Rock Samples Before and After Grouting

**Figure 4:**
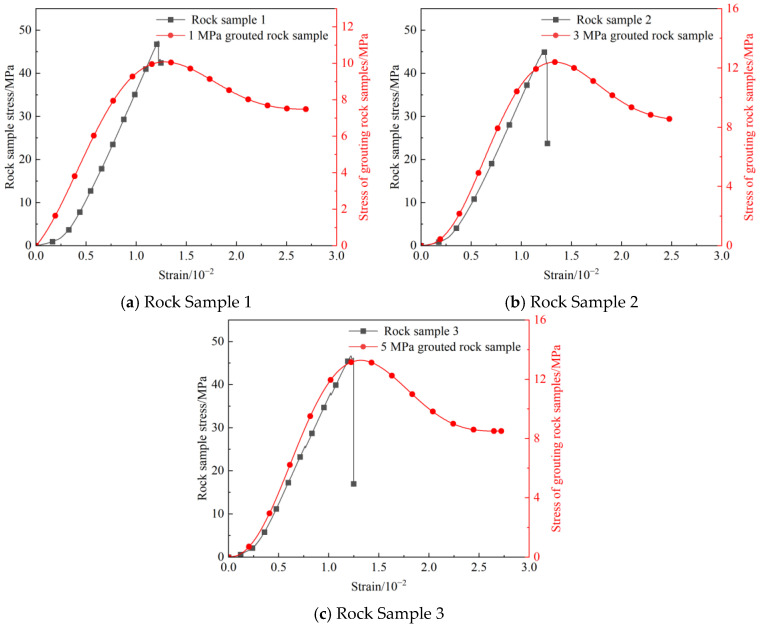
Stress–strain curves of rock samples before and after grouting.

Stress–strain curves were plotted for comparison by selecting data from a single representative specimen under each of the three different grouting pressures to analyze the effect of the grouting process on the changes in the stress–strain curves of the rock specimens, as shown in Figure 4. By conducting a comparative analysis of the stress–strain curves of rock samples under different grouting pressures versus their corresponding original rock samples, a significant effect of the grouting reinforcement process on the mechanical properties of the rock mass can be observed.

In Figure 4a, the stress–strain curve of Rock Sample 1 drops almost vertically and steeply after reaching the peak stress, with the post-peak ductility nearly disappearing. In contrast, after grouting at 1 MPa, the stress increase with strain during the elastic stage of the rock sample becomes more gradual, and the post-peak stress shows a slow declining trend, exhibiting clear ductile failure characteristics.

In Figure 4b, the post-peak curve of Rock Sample 2 plummets steeply, with prominent brittle characteristics; after grouting at 3 MPa, the elastic stage of the rock sample is similar to that before grouting, but the post-peak stress decline slope is significantly reduced. The curve transitions from a “sharp drop” to a “gentle decline”, making the ductility improvement effect visually apparent.

In Figure 4c, the stress of Rock Sample 3 drops rapidly after the peak, exhibiting typical brittle failure. After grouting at 5 MPa, the rock sample behaves similarly to the one grouted at 3 MPa, and its post-peak stress decline process is even more gradual. Furthermore, after the strain exceeds the peak point, the grouted rock sample can still sustain deformation under a relatively high stress level. These differences in curve morphology indicate the improvement effect of grouting reinforcement on the mechanical behavior and failure mode of the rock samples.

**Figure 5:**
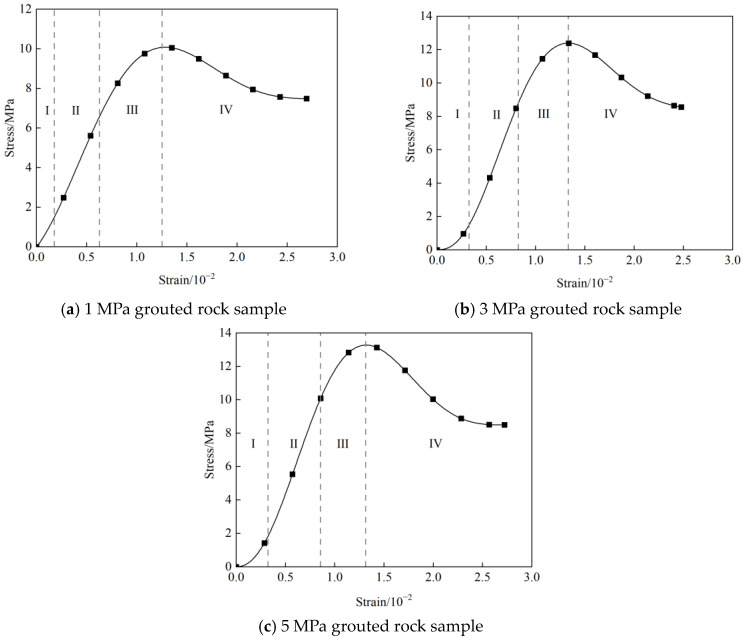
Stress–strain curves of grouted rock samples.

Figure 5 shows the complete stress–strain curves of the grouted rock samples under different grouting pressures during uniaxial compression, analyzed by dividing them into four stages.

Stage I (Initial compaction stage): All grouted rock samples showed slight nonlinear characteristics. In this stage, due to incomplete contact between the rock sample and the testing machine, the slope of the curve gradually increased from a minimum, indicating the progressive closure of micro-cracks and pores. The differences in this stage do not reflect the load-bearing characteristics of the rock samples.

Stage II (Linear elastic stage): The rock samples entered the stage of elastic deformation, and the stress–strain relationship exhibited good linear characteristics. The slope of the curve for the 1 MPa grouted rock sample remained constant until the stress reached approximately 65% of the peak strength. For the 3 MPa grouted rock sample, the span of the elastic stage was slightly larger than that of the 1 MPa sample, while it was longest for the 5 MPa sample. However, a slight nonlinear deviation had already appeared in the 5 MPa sample when the stress reached 70% of the peak strength.

Stage III (Plastic yielding stage): After the stress exceeded the elastic limit, the rock samples entered the stage of plastic deformation. The grouted rock samples exhibited obvious strain hardening characteristics in this stage, with the curve slope gradually decreasing until reaching the peak strength. The three rock samples followed roughly the same trend in this stage, with the 1 MPa grouted rock sample having the smallest peak strength, and the peak strengths of the 3 MPa and 5 MPa grouted rock samples were close.

Stage IV (Post-peak softening stage): The curve maintains a relatively high residual stress after the peak, which significantly improves the post-peak characteristics of the grouted rock samples, transforming the failure mode from brittle to ductile.

### 3.2. Compressive Strength and Elastic Modulus of Grouted Rock Samples

**Figure 6:**
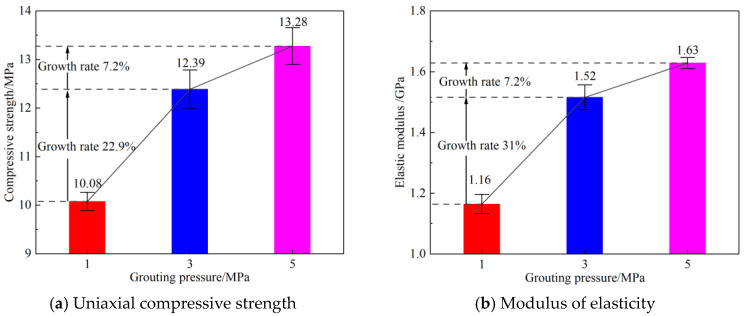
Compressive strength and elastic modulus of grouted rock samples.

Figure 6 indicates that grouting reinforcement altered the mechanical properties of the rock specimens. However, the reinforcement effects differed under various grouting pressures. At a grouting pressure of 1 MPa, the uniaxial compressive strengths of the three rock specimens were 9.876 MPa, 10.123 MPa, and 10.241 MPa, respectively, with an average peak strength of 10.08 MPa. At 3 MPa, the uniaxial compressive strengths of the three specimens were 11.985 MPa, 12.412 MPa, and 12.773 MPa, respectively, yielding an average peak strength of 12.39 MPa—a significant increase of 22.9% compared to the 1 MPa condition. At 5 MPa, the uniaxial compressive strengths of the three specimens were 12.891 MPa, 13.305 MPa, and 13.644 MPa, respectively, with an average peak strength of 13.28 MPa, representing a 7.2% increase relative to the 3 MPa condition. The elastic modulus also increased with higher grouting pressure. At 1 MPa, the elastic moduli of the three specimens were 1.125 GPa, 1.168 GPa, and 1.187 GPa, respectively, corresponding to an average of 1.16 GPa. At 3 MPa, the elastic moduli were 1.475 GPa, 1.53 GPa, and 1.555 GPa, respectively, with an average of 1.52 GPa—a substantial increase of 31% compared to the 1 MPa condition. At 5 MPa, the elastic moduli were 1.61 GPa, 1.635 GPa, and 1.645 GPa, respectively, averaging 1.63 GPa, which is a 7.2% increase over the 3 MPa condition. Increasing the grouting pressure enhanced both the compressive strength and elastic modulus of the specimens. The trends for these two parameters were generally consistent, and their magnitudes of improvement showed a positive correlation. A significant leap in peak strength and elastic modulus occurred when the pressure increased from 1 MPa to 3 MPa; however, the improvement tended to plateau when the pressure rose from 3 MPa to 5 MPa. This indicates that the improvement in mechanical properties follows a pattern of “diminishing returns” with increasing pressure.

### 3.3. Energy Evolution During Compression of Grouted Rock Samples

During the compression process of rock samples, a portion of the mechanical potential energy is converted into elastic strain energy stored within the sample. According to the first law of thermodynamics, another portion of energy is dissipated when the rock sample fails [29]. Therefore, the mechanical potential energy is ultimately transformed into elastic strain energy and dissipated energy, as expressed in Equation (1).

(1) $$U^{m}=U^{e}+U^{d}$$

When external influences are disregarded, the mechanical potential energy is equivalent to the total strain energy of the rock sample, as shown in Equations (2) and (3).

(2) $$U^{m}=U$$

(3) $$U=U^{e}+U^{d}$$

where $U^{m}$ is the mechanical potential energy of the rock sample, kJ/m^3^; U is the total strain energy of the rock sample, kJ/m^3^; $U^{e}$ is the elastic strain energy of the rock sample, kJ/m^3^; and $U^{d}$ is the dissipated energy of the rock sample, kJ/m^3^.

Based on the stress–strain curve of the rock sample, the calculation formulas for the strain energy of the rock sample during the compression process are given by Equations (4) and (5). Equation (5) for the elastic strain energy assumes linear elasticity throughout loading and is therefore not valid during plastic deformation [30,31].

(4) $$U=\int_{0}^{\varepsilon }\sigma d\varepsilon$$

(5) $$U^{e}=\frac{1}{2}\sigma \varepsilon^{e}=\frac{1}{2E_{u}}\sigma^{2}$$

where σ is the stress of the rock sample, MPa; ε is the strain of the rock sample; and $E_{u}$ is the elastic modulus of the rock sample, MPa.

**Figure 7:**
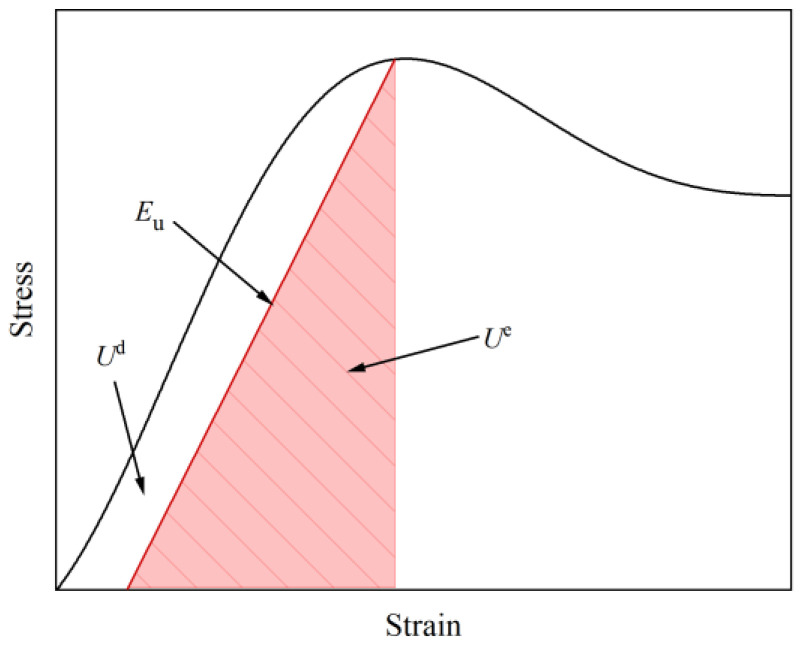
Energy evolution patterns.

The energy evolution during uniaxial compression of the grouted rock samples is shown in Figure 7. The area enclosed by the stress–strain curve and the x-axis represents the total strain energy of the rock sample; the area represented by a triangle with stress as its height and strain as its base indicates the elastic strain energy accumulated in the rock sample under that stress–strain condition; the difference between these two areas represents the energy dissipated by the rock sample.

**Figure 8:**
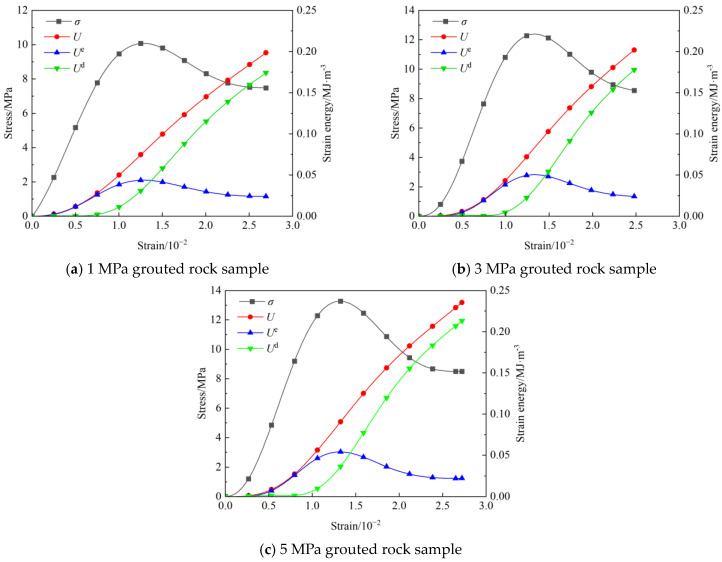
Energy evolution during compression of grouted rock samples.

Figure 8 illustrates the evolution of stress and energy with strain during the loading process of rock samples under different grouting pressures. The energy response curves in the figure are divided into total strain energy, elastic strain energy, and dissipated energy. Regarding total strain energy, the energy accumulation characteristics of the grouted rock samples are influenced by the grouting pressure. Under the three grouting pressures, the energy accumulation of the rock samples in the initial loading stage is relatively slow, with total strain energies of 0.199 MJ/m^3^, 0.202 MJ/m^3^, and 0.235 MJ/m^3^, respectively. The total strain energy of the 3 MPa grouted sample shows only a slight increase compared to the 1 MPa grouted sample, while the 5 MPa grouted sample exhibits the highest total strain energy. This indicates that, under certain conditions, grouting pressure is positively correlated with energy accumulation.

The energy dissipation process exhibits distinct staged characteristics. In the initial stage, the energy dissipation of the rock samples under the three grouting pressures is almost zero, with an extremely low dissipation rate. Upon entering the plastic stage, damage begins to develop, and the energy dissipation rate gradually increases. In the post-peak stage, energy dissipation rises sharply, primarily converting into the surface energy required for crack propagation. At this point, the dissipated energy of the grouted rock samples increases further. Under the three grouting pressures, the dissipated energies of the rock samples are 0.174 MJ/m^3^, 0.177 MJ/m^3^, and 0.213 MJ/m^3^, respectively, accounting for 87.4%, 87.6%, and 90.6% of the total strain energy. The 5 MPa grouted rock sample exhibits the highest dissipated energy.

Regarding the evolution of elastic strain energy, rock samples under the three grouting pressures all exhibited a common trend of initially increasing, then slowly decreasing, while retaining a certain amount of residual elastic strain energy. The peak elastic strain energies were 0.044 MJ/m^3^, 0.051 MJ/m^3^, and 0.054 MJ/m^3^, respectively. The 3 MPa grouted rock sample increased by 15.9% compared to the 1 MPa grouted rock sample, whereas the 5 MPa grouted rock sample increased by only 5.9% compared to the 3 MPa grouted rock sample. The differences in residual elastic strain energy under different grouting pressures were not significant. The analysis results indicate that increasing the grouting pressure helps enhance the ability of rock samples to store elastic strain energy before failure, with the improvement effect being most pronounced when increasing from 1 MPa to 3 MPa.

### 3.4. Toughness and Brittleness of Grouted Rock Samples

**Figure 9:**
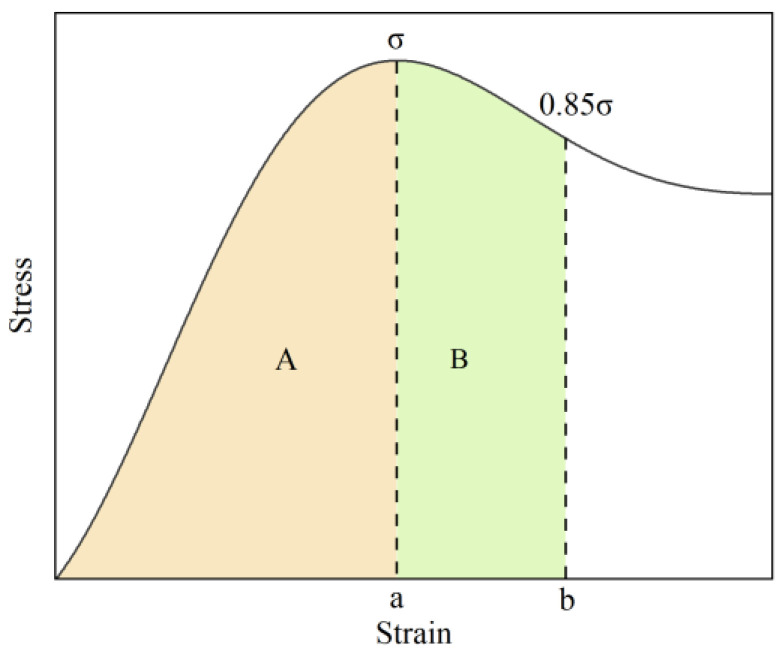
Schematic diagram of toughness and brittleness indices.

Toughness reflects the ability of a rock sample to resist deformation or absorb energy during the deformation and failure process. The higher the toughness index, the lower the risk of brittle fracture; brittleness is characterized by sudden failure after reaching a critical load, and its brittleness index is negatively correlated with the toughness index [32]. Based on the uniaxial compression stress–strain curves, as shown in Figure 9, the segment from the starting point to the strain corresponding to 0.85 times the peak stress is taken as the calculation interval. The toughness index and brittleness index are calculated using Equations (6) and (7), respectively. Table 4 presents the calculation results and average values of the toughness and brittleness of rock samples under different grouting pressures.

(6) $$T=\frac{A+B}{A}=\frac{\int_{0}^{b}\sigma d\sigma }{\int_{0}^{a}\sigma d\sigma }$$

(7) $$D=\frac{A}{B}=\frac{\int_{0}^{a}\sigma d\sigma }{\int_{a}^{b}\sigma d\sigma }$$

where *T* is the toughness index; *D* is the brittleness index; and *A* and *B* are both the areas enclosed by the curve and the X-axis, with *A* corresponding to the segment from the starting point to the peak stress point a, and *B* corresponding to the segment from the peak stress point a to the 0.85 times peak stress point b.

**Figure 10:**
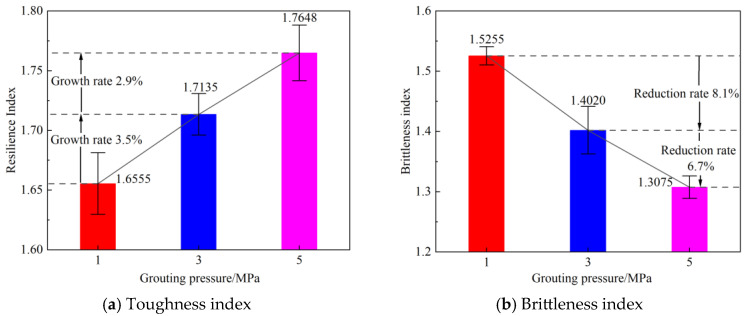
Toughness and brittleness indices of grouted rock samples under different pressures.

Figure 10 illustrates the variation in the average toughness index and brittleness index of the rock specimens under different grouting pressures. Regarding toughness, the grouting pressure exerted a significant influence on the toughness characteristics of the specimens. The average toughness index of the specimens grouted at 1 MPa was 1.6555. When the grouting pressure was increased to 3 MPa, the average toughness index rose to 1.7135, representing a significant increase of 3.5% compared to the average under the 1 MPa condition. The average toughness index for specimens grouted at 5 MPa was 1.7648, the highest among the three pressures. However, it increased by only 2.9% compared to the average at 3 MPa, indicating a slowdown in the rate of improvement. This trend suggests that appropriately increasing the grouting pressure can effectively enhance the overall deformability of the rock specimens.

In terms of brittle characteristics, grouting demonstrated a significant inhibitory effect on the brittleness of the specimens. The average brittleness index was 1.5255 for specimens grouted at 1 MPa. It decreased to an average of 1.4020 at 3 MPa, a reduction of 8.1%. The average brittleness index further declined to 1.3075 at 5 MPa, which is 6.7% lower than the average at 3 MPa, showing a weakened rate of decrease.

The reduction in the brittleness index is mainly reflected in the following two aspects: first, the strain at failure for the grouted rock samples increases significantly; second, the failure mode shifts from brittle failure to ductile failure. This is basically consistent with the variation characteristics of the stress–strain curves of the rock samples before and after grouting shown in Figure 4, fully demonstrating that increasing the grouting pressure helps enhance the ductile failure of the rock mass.

The comprehensive analysis indicates that grouting reinforcement effectively promotes the transition of the rock samples’ mechanical behavior from brittle to ductile. Among the different grouting pressures, the 3 MPa grouting pressure achieves significant effects both in enhancing toughness and in suppressing brittleness, demonstrating superior comprehensive improvement effectiveness.

### 3.5. Ductility of Grouted Rock Samples

**Figure 11:**
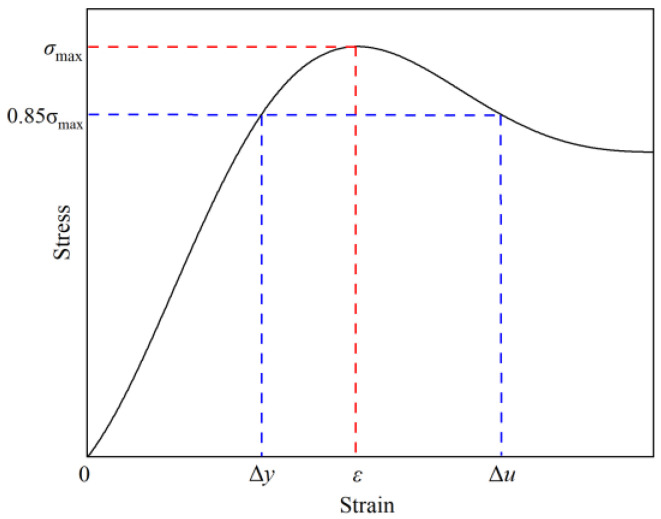
Characteristic points of the ductility index.

Ductility refers to the deformation capacity of a rock sample from the onset of yielding until it reaches its maximum load-bearing capacity or a period thereafter during which the bearing capacity does not significantly decline. For grouted rock samples, it is an important indicator for analyzing their ductility enhancement effect [33]. In this paper, the ductility indicator is used to represent this, calculated using two specific points on the stress–strain curve: one at 85% of the peak stress before the peak and one at 85% of the peak stress after the peak, as shown in Figure 11.

The damage process of grouted rock samples can be divided into three stages: initial crack initiation, stable development, and unstable failure. When the stress reaches 85% of the peak stress, the material enters the stage of unstable crack development, where cracks propagate rapidly and lead to final failure. Selecting this characteristic point can reflect the ductility performance of the material during the significant plastic deformation stage, avoiding assessment bias that is either too early (elastic stage) or too late (complete failure). Selecting the point corresponding to 85% of the peak stress as the characteristic point for calculation can avoid data dispersion caused by the sharp stress drop in the post-peak stage, and the measurement by the experimental equipment is more stable during this stage, ensuring the reproducibility and accuracy of the results [34,35,36].

It can be intuitively seen from the figure that the ductility capacity of the rock samples is related to the slopes before and after the peak of the stress–strain curve. Both the pre-peak and post-peak characteristic points correspond to the 85% peak stress point. The ductility index μ is calculated using Equation (8). Table 5 shows the calculation results and average values of the ductility index of rock samples under different grouting pressures.

(8) $$\mu =\frac{\Delta u}{\Delta y}$$

where Δu corresponds to the strain at 85% of the peak stress after the peak; Δy corresponds to the strain at 85% of the peak stress before the peak.

**Figure 12:**
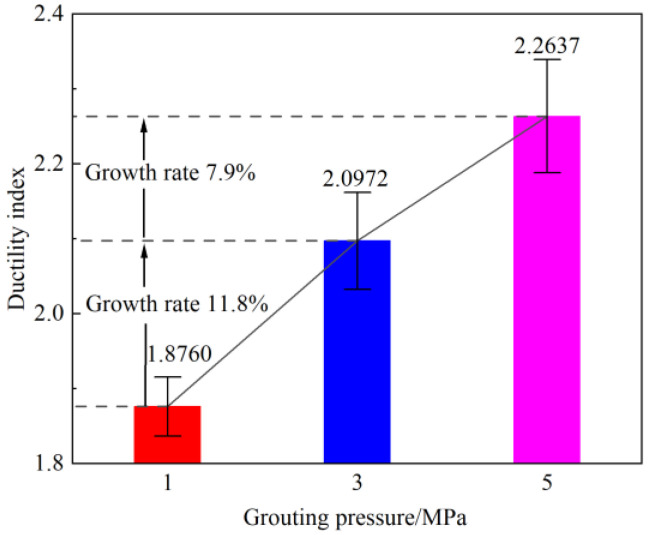
Ductility index of grouted rock samples under different pressures.

Figure 12 presents the variation in the average ductility index of rock specimens under different grouting pressures. Grouting significantly enhanced the deformation capacity of the rock specimens. Specifically, the average ductility index of specimens grouted at 1 MPa was 1.8760; at 3 MPa, it increased to 2.0972, representing an 11.8% improvement over the 1 MPa condition; at 5 MPa, the average ductility index further rose to 2.2637, corresponding to a 7.9% increase relative to the 3 MPa condition. This indicates that increasing the grouting pressure can markedly improve the ductility of the rock samples, and the higher the pressure, the stronger the overall deformation capacity of the rock samples. Although raising the grouting pressure enhances the deformation capacity of the rock samples, the rate of increase tends to slow down as the pressure rises. The 3 MPa grouting pressure achieved a relatively significant ductility improvement within a reasonable range, demonstrating high improvement efficiency.

## 4. Scanning Electron Microscopy Microstructure of Grouted Rock Samples

### 4.1. Microstructural Composition of the Grout Matrix and Rock Sample Matrix

**Figure 13:**
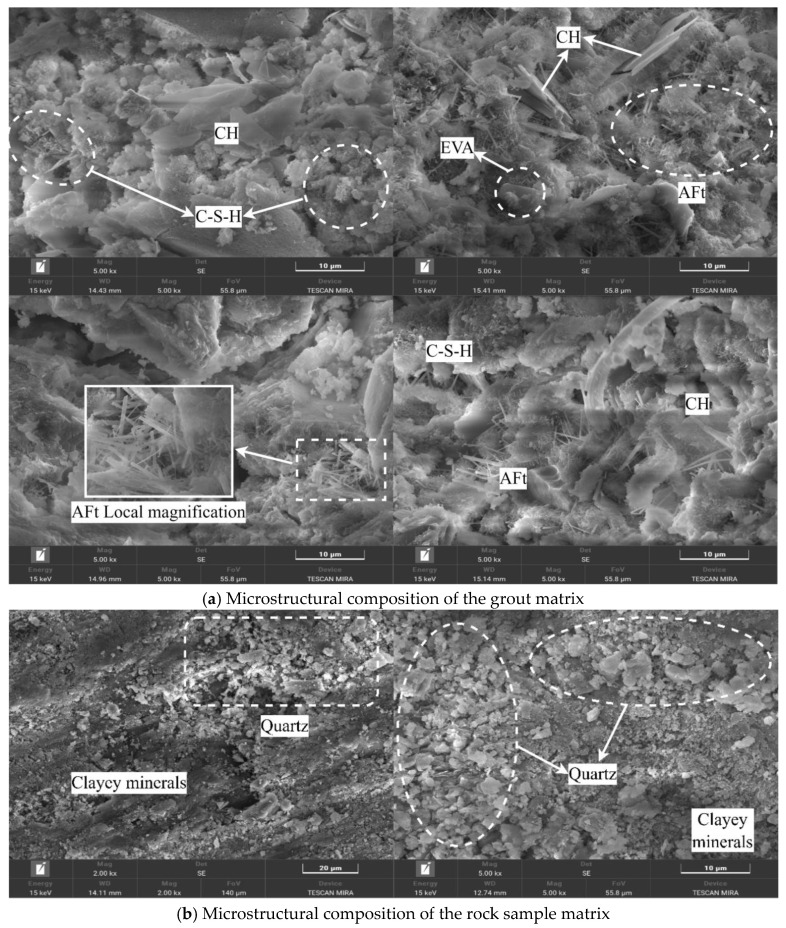
Microstructural composition of the grout matrix and rock sample matrix.

Figure 13 shows the characteristic map of the microscopic composition of the grout matrix and the rock sample matrix, allowing for clear identification of the core microscopic components and distribution patterns of the two matrices.

As shown in Figure 13a, the core microscopic components of the grout matrix are cement hydration products, primarily including C-S-H (calcium silicate hydrate) gel, CH (calcium hydroxide) crystals, AFt (ettringite) crystals, and others. Among these, the C-S-H gel primarily exhibits a flocculent morphology and is widely distributed throughout the grout matrix, interweaving to form a continuous spatial network structure that constitutes the cementitious skeleton of the grout matrix; the CH crystals, on the other hand, display a regular tabular morphology, embedded within the C-S-H gel network in a dispersed manner, with clear crystal edges and relatively uniform sizes, and no significant agglomeration is observed; the AFt crystals exist in an acicular or rod-shaped morphology, often interspersed between the C-S-H gel and CH crystals, with some AFt crystals interconnecting, further enhancing the integrity of the hydration product system. The hydration products are distributed harmoniously, with no apparent blank areas or missing components, indicating sufficient hydration reaction of the grout and providing support for the structural strength of the grout matrix.

The fact that the specimen structure possesses good compactness is also closely related to the role of EVA latex particles. They first adsorb onto the surface of cement particles and form a thin film, then gradually fill the capillary pores within the hardened grout body. As the cement hydration reaction continues, this EVA film, together with the hydration products, builds a three-dimensional network structure. This structure acts as a “bridge” connecting the components, not only optimizing the performance of the interfacial transition zone but ultimately promoting the formation of a continuous and integrated overall structure in the rock sample.

As shown in Figure 13b, the core components of the rock sample matrix are natural minerals, primarily consisting of two types: quartz and clay minerals. Quartz mainly exists in granular form with smooth surfaces and distinct, angular edges. The particle sizes show little variation, and they are mostly distributed as independent individuals, forming the rigid skeleton of the rock sample matrix. Clay minerals, on the other hand, exhibit flocculent or platy morphologies, primarily filling the gaps between quartz particles. Some clay minerals adhere to the surfaces of quartz particles, forming thin coating layers. The distribution of clay minerals is relatively uniform, with no localized aggregation or absence observed. The rigid characteristics of quartz particles and the flexible filling of clay minerals complement each other, collectively constituting the natural mineral structure of the rock sample matrix. However, there are no apparent chemical reaction products between the two minerals; they bond only through physical contact, which differs from the “hydration product cementation” bonding method found in the grout matrix.

### 4.2. Microstructure of the Interface Between the Grout Matrix and the Rock Sample Matrix

**Figure 14:**
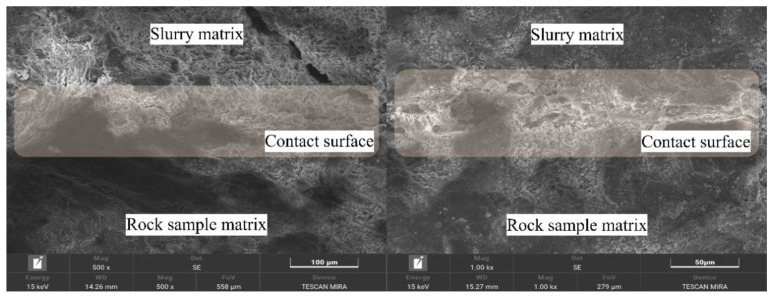
Microstructure of the interface between the grout matrix and the rock sample matrix.

Figure 14 presents the micromorphology of the contact interface between the grout matrix and the rock sample matrix, showing the structural connection state of the two matrices at the bonding interface. It allows for a clear visual distinction among the “grout matrix zone,” the “contact interface zone,” and the “rock sample matrix zone”. From the perspective of the overall interface structure, no obvious fracture gaps or large-area blank separations are observed at the interface. The grout matrix and the rock sample matrix exhibit a “progressive transition” characteristic. The grout matrix does not contact the rock sample matrix along a flat plane; instead, parts of it extend into the gaps of the rock sample matrix, forming microscopic “embedded segments.” This morphology indicates that during the grouting process, the grout could penetrate into the microscopic pores of the rock sample matrix. By providing a foundation for interface bonding through physical interlocking, this mechanism avoids the occurrence of mere “surface contact” at the interface, thereby enhancing the bonding stability.

Near the interface, the grout matrix maintains a dense cementitious structure with no apparent loosening or delamination. The cementitious materials extend continuously into the gaps of the rock sample matrix; some of these materials even envelop fine particles on the surface layer of the rock sample matrix, forming a local “slurry-particle” encapsulation structure, which further strengthens the connection between the two sides. The gaps between particles in the rock sample matrix near the interface are fully filled by the grout. The originally independently stacked mineral particles achieve indirect cementation through the grout’s cementitious materials, rather than mere physical contact. Moreover, no particle detachment or structural damage caused by grout penetration is observed on the surface layer of the rock sample matrix, indicating that the interfacial interaction between the grout and the rock did not compromise the integrity of the rock sample’s own microstructure.

Furthermore, a very small number of microscopic voids are observed locally at the contact interface. These voids are mostly concentrated at the junctions between larger particles of the rock sample matrix and the grout. This may be due to the irregular shapes of the rock particles making it difficult for the grout to achieve complete filling. However, the void sizes are extremely small and non-interconnected, and they do not affect the overall bonding state of the interface. This overall reflects that the grout matrix and the rock sample matrix are capable of forming a relatively stable interfacial bond.

### 4.3. Comparison of Microstructural Features of Fractures in Grouted Rock Samples

**Figure 15:**
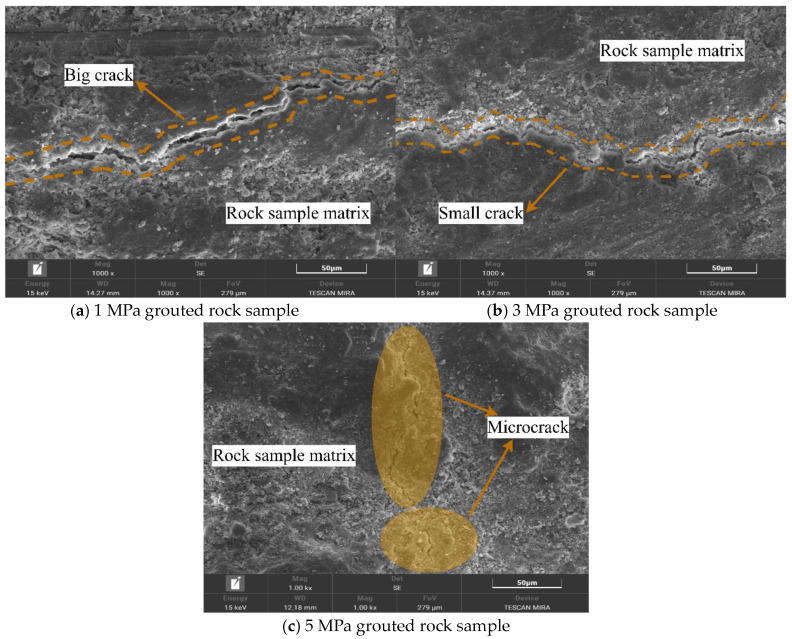
Micromorphology of grouted rock sample matrix cracks.

Figure 15 shows the micromorphology of cracks within the rock sample matrix under different grouting pressures. Through the comparison of multiple observation zones and in conjunction with key features such as “major cracks,” “small cracks,” and “microcracks,” it clearly presents the influence of grouting pressure on the development, distribution, and scale of cracks inside the rock sample matrix. All cracks are associated with the mineralogical structure of the rock sample matrix.

When the grouting pressure is low (1 MPa), distinct “major cracks” can be observed in the rock sample matrix. These cracks predominantly propagate along the gaps between the natural mineral particles of the rock sample. They are relatively long, traversing portions of the rock sample region, and exhibit a relatively wide crack width. Their edges are accompanied by irregular traces of mineral particle detachment. A small number of “small cracks” are also distributed around the major cracks. These small cracks often extend from the ends of the major cracks towards the surrounding mineral particles. They are relatively short and mostly terminate at particle boundaries. This reflects that under low pressure, the penetration and filling effect of the grout on the rock sample matrix is limited. The inherent structural defects of the rock sample itself (such as particle gaps and natural micro-cracks) are not effectively covered. Furthermore, it is even possible that localized stresses during the grouting process cause these inherent defects to expand into major and small cracks, thereby damaging the structural integrity of the rock sample matrix.

As the grouting pressure increases, the crack morphology in the rock sample matrix undergoes a significant transformation. The number of major cracks decreases substantially, with only sporadic occurrences in areas where mineral particle distribution is locally uneven. These are replaced by numerous “small cracks” and “microcracks.” These microcracks are extremely fine in width, mostly distributed along the interfaces between clay minerals and rigid particles within the rock sample matrix, and are relatively short and appear discontinuous, showing no obvious through-going character. Some microcracks also form fine fissures surrounding individual mineral particles, without extending to the surroundings. This indicates that under higher pressures (3 MPa, 5 MPa), the grout can more fully penetrate into the microscopic pores and particle gaps of the rock sample matrix. The filling effect of the grout inhibits the propagation of the rock’s inherent defects into major and small cracks, resulting only in a small number of microcracks that have a minor impact on the overall structure, due to the microscopic inhomogeneity of the rock’s mineral composition.

Overall, Figure 15 clearly demonstrates the pattern that “with increasing grouting pressure, the cracks in the rock sample matrix shift from being dominated by major cracks, accompanied by small cracks, towards being dominated by microcracks with a reduction in major cracks.” Furthermore, all cracks within the rock sample matrix are highly associated with its own mineralogical structure (particle gaps, mineral interfaces). This characteristic provides a microstructural basis for subsequently optimizing grouting pressure, enhancing the filling effect of the grout on rock sample gaps, thereby reducing major cracks in the rock sample matrix and improving the overall stability of the grout matrix-rock sample matrix composite.

### 4.4. Comparison of Micromorphology of Fracture Contact Interfaces in Grouted Rock Samples

**Figure 16:**
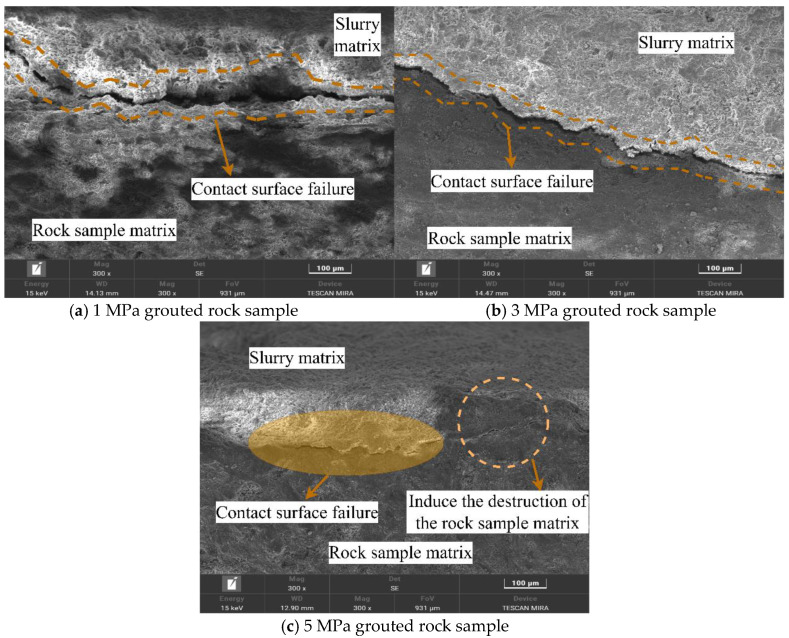
Micromorphology of grouted rock sample contact surface.

Figure 16 presents the failure characteristics of the “grout matrix–rock matrix” contact interface under different grouting pressures. By examining the “grout matrix zone,” “contact interface failure zone,” and “rock matrix zone,” the influence of grouting pressure on the failure morphology, extent of damage at the contact interface, and the effect of the grouting process on internal damage to the rock specimen is clearly illustrated. At a lower grouting pressure (1 MPa), the “contact interface failure zone” exhibited significant debonding, with a pronounced separation gap between the grout matrix and rock matrix. In some areas, complete failure was observed, evidenced by clear detachment marks on the grout matrix. As the grouting pressure increased, the failure morphology at the interface improved noticeably. The extent of debonding in the “contact interface failure zone” was reduced, partial adhesion was preserved between the grout and rock matrices, and a distinct cementitious transition layer became apparent.

**Figure 17:**
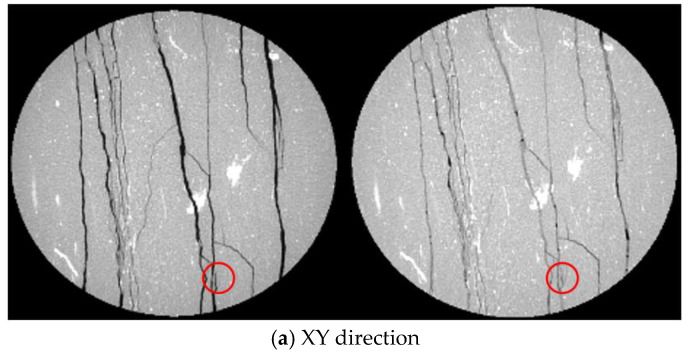

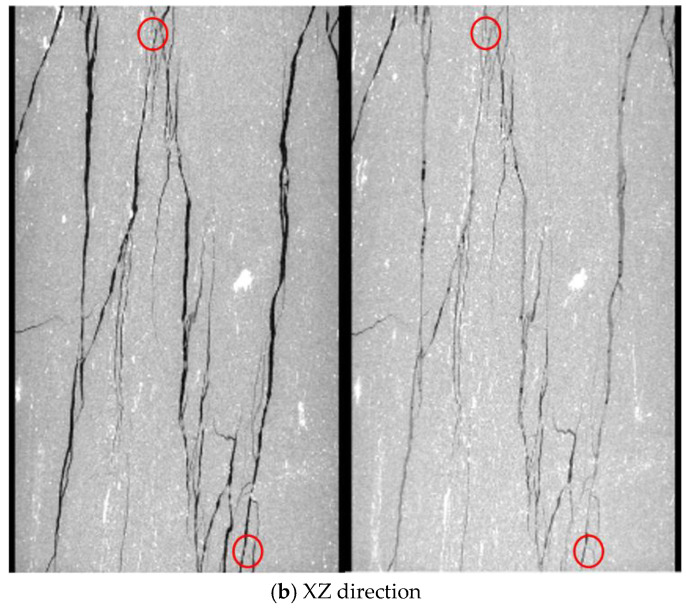
CT scan image slices of rock samples before and after grouting.

However, a higher grouting pressure (5 MPa) can induce new cracks within the rock matrix before the uniaxial compression test loading. Fine pre-existing cracks within the fractured rock matrix can extend further into the interior under high grouting pressure, causing slight loosening of some rock particles due to crack propagation. A comparison of CT slices before and after grouting supports this observation. In Figure 17a,b, the left side shows CT slices before grouting, and the right side shows slices after grouting at 5 MPa. As can be seen from Figure 17, under a grouting pressure of 5 MPa, new and identifiable mesoscopic cracks have appeared inside the rock matrix, indicating that the grouting process itself has caused new damage to the inherent structure of the rock.

A comprehensive analysis of the relationship between the micromorphological characteristics of rock specimens under different grouting pressures (illustrated in Figure 13, Figure 14, Figure 15, Figure 16 and Figure 17) and mechanical properties revealed the following correspondences:

Correspondence between energy dissipation trends and SEM microcrack patterns: As the grouting pressure increased from 1 MPa to 3 MPa and further to 5 MPa, the failure mode of the rock specimens transitioned from rapid coalescence of a few dominant macroscopic cracks to diffuse initiation and stable propagation of numerous microcracks. This transition is reflected in the energy evolution curves by a reduced post-peak energy dissipation rate and an increased proportion of total dissipated energy. SEM images (Figure 15) substantiate this mechanism: under 1 MPa, failure was dominated by wide primary cracks, whereas under 3 MPa and 5 MPa, primary cracks were significantly reduced and replaced by dense networks of microcracks distributed along mineral boundaries. This indicates that increasing grouting pressure alters the sites of crack initiation and propagation paths, thereby reshaping the spatiotemporal distribution characteristics of energy dissipation.

Correspondence between ductility enhancement and grout penetration characteristics revealed by CT: The ductility average index exhibited a continuous increase with rising grouting pressure (1.8760 ⟶ 2.0972 ⟶ 2.2637). Correspondingly, the 3D CT reconstruction models (Figure 12) clearly demonstrate that at 3 MPa, the degree of grout filling within micropores and interparticle voids was substantially higher than that at 1 MPa, forming a more continuous and dense grout vein network that effectively enhanced the overall deformation compatibility of the composite structure. At 5mpa, although the slurry distribution further expanded, new micro-cracks appeared inside the rock matrix (Figure 17). Therefore, the mechanism underlying ductility enhancement can be summarized as follows: an appropriate grouting pressure improves grout penetrability and filling density, thereby optimizing the stress transfer path and damage degree of the composite material.

## 5. Numerical Simulation of Fracture Crack Evolution in Grouted Rock Samples

### 5.1. Establishment of a Uniaxial Compression Numerical Model

#### 5.1.1. Establishment of CT-Reconstructed Rock Specimen Models

**Figure 18:**
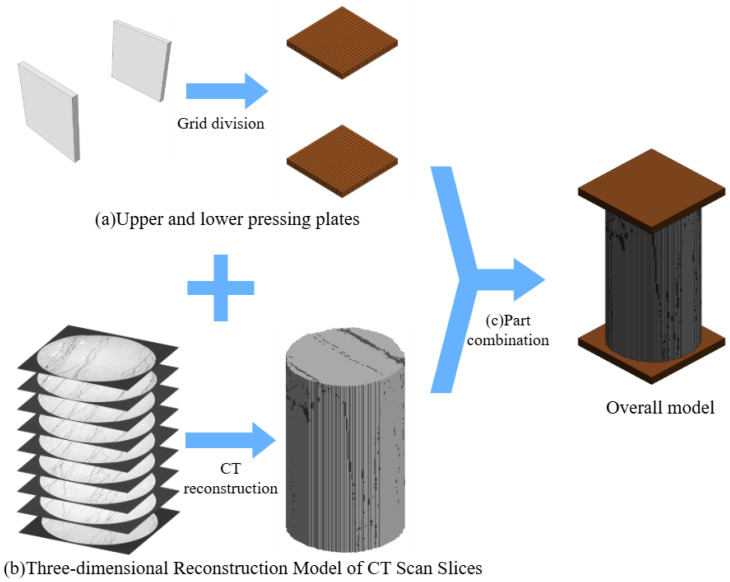
Three-dimensional reconstruction model of CT scan slices.

To ensure the reliability of the numerical simulation, a coupled modeling approach using ABAQUS and LS-DYNA was adopted, leveraging the advantages of geometric pre-processing and explicit dynamic analysis. ABAQUS provides powerful geometric modeling and meshing capabilities, making it particularly suitable for the preliminary construction of complex structures, whereas LS-DYNA excels in deformation and material failure analysis, rendering it highly appropriate for simulating the failure behavior of rock-like materials under uniaxial compression. In ABAQUS/CAE, the upper and lower loading platens were modeled as rigid cuboids with side lengths of 60 mm and a height of 5 mm. A hexahedral structured mesh was employed to enhance computational efficiency and numerical stability. A uniform element size of 0.8 mm was selected to capture stress concentrations and crack initiation within reasonable computational resources. Each platen comprised approximately 1200 elements, achieving a balance between computational accuracy and efficiency. Following meshing, the model was exported in a universal format and imported into LS-PrePost for material property assignment, contact definition, and the configuration of boundary and loading conditions. The final platen model, characterized by regular geometry and high mesh quality, is shown in Figure 18a, establishing a solid foundation for the subsequent uniaxial compression simulation.

To accurately characterize the spatial distribution of the grout after injection and its influence on the macroscopic mechanical behavior of the rock specimen, a high-fidelity numerical model was constructed based on industrial CT scan data. By employing image processing and model reconstruction techniques, the heterogeneous internal structure was directly translated into a numerical model, significantly enhancing the realism of the simulation. The specific workflow is as follows: First, the original CT images were preprocessed. The raw DICOM-format data were voluminous—typically comprising over 2000 slices per specimen with a resolution of 2048 × 2048 pixels—imposing substantial demands on computational memory for direct reconstruction. To address this, a combined downsampling and region extraction strategy was adopted. The image sequence was subsampled at equal intervals along the Z-axis to 400 slices to preserve the primary features in the vertical direction. Subsequently, the resolution of each image was reduced to 200 × 200 pixels, with bicubic interpolation applied to maintain clarity. Finally, all images were sorted and indexed according to their actual scanning positions to establish a mapping relationship with the three-dimensional spatial coordinates. Following this, image thresholding was performed to differentiate material components. Leveraging the differences in X-ray absorption coefficients among materials in the CT images, iterative threshold segmentation was conducted to separate the rock matrix from the grout matrix, ultimately generating a sequence of binary images. Three-dimensional surface reconstruction was carried out using the classical Marching Cubes algorithm, which converted the binary image sequence into a continuous surface volumetric mesh model, as depicted in Figure 18b. Finally, the platen model and the three-dimensionally reconstructed rock specimen model were merged in LS-PrePost. Boolean operations were applied to trim the combined geometry into a cylindrical rock specimen shape, eliminating redundant external regions. The model was subsequently smoothed, defective nodes were removed, and mesh quality was optimized to ensure that element metrics satisfied the explicit calculation requirements of LS-DYNA. The final in situ CT reconstruction model is presented in Figure 18c.

**Figure 19:**
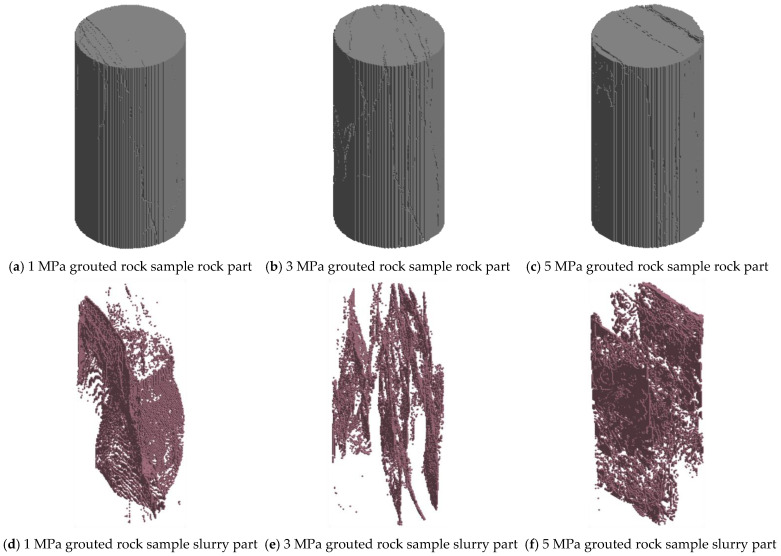
3D Reconstruction models of rock samples at different grouting pressures.

Furthermore, three-dimensional reconstruction models of rock specimens grouted under pressures of 1, 3, and 5 MPa are presented in Figure 19. The distribution of the rock matrix (gray) and the grout matrix (brown) clearly reproduces the characteristics of grout propagation within the fractures, providing a realistic geometric basis for subsequent numerical analysis.

#### 5.1.2. LS-DYNA Keyword Configuration

Keywords in LS-DYNA are the core elements defining all attributes of a numerical model. To ensure that the uniaxial compression simulation accurately reflects the mechanical behavior of both the rock sample and the grout material, the keyword settings were configured as follows.

Boundary Conditions: To simulate the constraints imposed by the testing machine platens, the lower platen was defined as fully fixed. First, a node set containing the bottommost nodes of the lower platen was created using *SET_NODE_LIST. Subsequently, a fixed boundary condition was applied to this node set by employing *BOUNDARY_SPC_SET, constraining all six degrees of freedom (DOFX, DOFY, DOFZ, DOFRX, DOFRY, DOFRZ) in the local coordinate system (with the displacement value set to 1).

Displacement Loading: Displacement control was adopted to match the physical testing procedure. The displacement–time curve for the upper platen was defined using *DEFINE_CURVE (prescribing a loading rate of 0.1 mm/s up to 15 mm over 0–150 s). The platens were modeled as rigid bodies using *MAT_RIGID. The top surface of the upper platen was selected as the loading surface via *SET_SEGMENT. Finally, the prescribed displacement curve was applied to the translational degree of freedom in the Z-direction (DOF = 3) of this rigid body segment set using *BOUNDARY_PRESCRIBED_MOTION_RIGID, thereby achieving a quasi-static compression simulation.

Contact Surface Definitions: The model primarily involved two types of contacts. For the contact between the platens and the rock sample, *CONTACT_AUTOMATIC_SURFACE_TO_SURFACE was used, with both static and dynamic friction coefficients set to 0.2. For the complex internal contact between the rock matrix and the grout material within the sample, *CONTACT_AUTOMATIC_SINGLE_SURFACE was employed, with the friction coefficient also set to 0.2.

Material Models: Three material keywords were utilized. The upper and lower platens were defined as rigid bodies using *MAT_RIGID. To describe the elastoplastic behavior and failure of the materials, *MAT_PLASTIC_KINEMATIC was combined with *MAT_ADD_EROSION. The latter used the maximum principal strain (MXEPS = 0.15) as the failure criterion to simulate element erosion and macroscopic crack propagation.

For the rock sample and grout matrix, the *MAT_RHT (*MAT_272) constitutive model was adopted. Through this detailed configuration of keywords, a numerical model was established that is capable of accurately capturing the mechanical response and failure process of both intact and grouted rock samples under uniaxial compression.

#### 5.1.3. RHT Constitutive Model

The RHT (Riedel–Hiermaier–Thoma) constitutive model is widely used in the dynamic behavior simulation of concrete and rock materials. This model can comprehensively consider pressure hardening, strain rate effect and damage accumulation [36]. The RHT model employs a Mie–Grüneisen equation of state to describe the pressure–volumetric strain relationship [37]. Its key parameters include material porosity, pressure, volume, and internal energy. The governing equation is given in Equation (9).

(9) $$P=\left\{\begin{array}{cc} \frac{1}{a}(A_{1}h+A_{2}h^{2}+A_{3}h^{3}+(B_{0}+B_{1}h)\rho_{0}e), & h>0\\ \frac{1}{a}(T_{1}h+T_{2}h^{2}+B_{0}\rho_{0}e), & h<0 \end{array}\right.$$

where h is the volumetric strain; $\rho_{0}$ is the initial density, kg/m^3^; e is the specific internal energy, J/kg; and $A_{1}$, $A_{2}$, $A_{3}$, $B_{0}$, $B_{1}$, $T_{1}$, $T_{2}$ are coefficients to be determined.

The RHT model characterizes material behavior through three key stress surfaces: the elastic yield surface delineates the elastic–plastic transition; the failure surface defines the maximum stress state before rupture; and the residual friction surface describes post-failure strength from fragment interactions. The failure surface is mathematically defined by Equation (10).

(10) $$\sigma_{f}=f_{C}\sigma_{TXC}{\left(\frac{P}{f_{c}}\right)}^{N}\left(1+C\ln E^{*}\right)R_{3}(\theta )$$

where $\sigma_{f}$ is the failure stress, MPa; $f_{C}$ is the uniaxial compressive strength, MPa; $\sigma_{TXC}$ is the equivalent stress intensity of the principal compression axis, MPa; p is the pressure, MPa; N is the pressure hardening index; C is the strain rate coefficient; $E^{*}$ is the strain rate; and $R_{3}\left(\theta \right)$ is the Lode angle factor.

In ANSYS/LS-DYNA, the RHT constitutive model is implemented via the *MAT_272 keyword. This model comprises a total of 37 parameters, among which five are fundamental physical parameters that can be directly measured or obtained (e.g., density, elastic modulus). Fourteen parameters are derived from the existing literature [38,39], while the remaining 18 parameters are relatively insensitive to the simulation outcomes and are therefore assigned the recommended default values. The specific values of these parameters are detailed in Table 6.

The compression strain rate index $\beta_{C}$ and tensile strain rate index $\beta_{t}$ listed in the table can be determined according to Equations (11) and (12), respectively:

(11) $$\beta_{C}=\frac{4}{20+3f_{c}}$$

(12) $$\beta_{t}=\frac{2}{20+f_{c}}$$

where $f_{C}$ is the compressive strength of the material, MPa.

Seven RHT constitutive model parameters require calibration: $A_{1}$, $A_{2}$, $A_{3}$, $B_{0}$, $B_{1}$, $T_{1}$, $T_{2}$. These parameters are calculated using Equation (13).

(13) $$\left\{\begin{array}{l} A_{1}=\rho_{0}c_{0}^{2}\\ A_{2}=\rho_{0}c_{0}^{2}(2s-1)\\ A_{3}=\rho_{0}c_{0}^{2}(3s^{2}-4s+1)\\ T_{1}=\rho_{0}c_{0}^{2}\\ B_{0}=B_{1}=2s-1\\ T_{2}=0 \end{array}\right.$$

where $A_{1}$, $A_{2}$, $A_{3}$ are Hugonoit polynomial coefficients; $c_{0}$ is the sound velocity in the material at zero pressure, m/s; $B_{0}$, $B_{1}$, $T_{1}$, $T_{2}$ are state equation parameters; and s is an empirical parameter.

The failure surface is defined by parameters A and N, whose relationship is given by Equation (14) and is subject to the constraint 3*p*_0_ ≥ *F* (compressive strength) [40].

(14) $$\sigma_{f}^{*}(p_{0},F_{r})=A{\left(p_{0}-F_{r}/3+{\left(A/F_{r}\right)}^{-1/N}\right)}^{N},\;3p_{0}\geq F_{r}$$

where $\sigma_{f}^{*}\left(p_{0},F_{r}\right)$ is the normalized strength relative to compressive strength, MPa; $F_{r}$ is the dynamic strain rate intensification factor; and $p_{0}$ is the normalized pressure, MPa.

The ratio Q between the tensile and compressive meridian radii in the RHT model is determined by the linear Equation (15). The coefficients for this equation were adopted from the experimental fitting results of prior research [41]:

(15) $$Q\;=\;Q(p_{0})\;=\;Q_{0}\;+\;Bp_{0}$$

where $Q_{0}$ is the tensile–compressive meridian ratio parameter and B is the Lode angle correlation coefficient.

Since the final simulation procedure involves three different grouting pressures, the RHT constitutive parameters were modified to distinguish the properties of the grout material under each pressure condition. The influence of grouting pressure on the grout’s microstructure is such that higher pressure results in a denser material with reduced initial porosity. Consequently, this enhances the macroscopic strength (reflected in the compressive and tensile strengths), stiffness (reflected in the shear modulus), and compressibility (reflected in the equation-of-state parameters *A*_1_, *A*_2_, *A*_3_ and the compaction pressure). Furthermore, the damage evolution parameter was adjusted to represent the material’s ability to withstand greater deformation before failure. The adjustments to the key parameters were based on the principle that increased grouting pressure leads to greater material densification, reduced porosity, and thus improved strength, stiffness, and damage resistance. Table 7 presents the key parameter values for grouting pressures of 1 MPa, 3 MPa, and 5 MPa, ensuring the rationality of simulating the grout’s behavior under different pressure conditions.

### 5.2. Analysis of Numerical Simulation Results for Grouted Rock Samples

#### 5.2.1. Analysis of Mechanical Properties of Grouted Rock Samples

**Figure 20:**
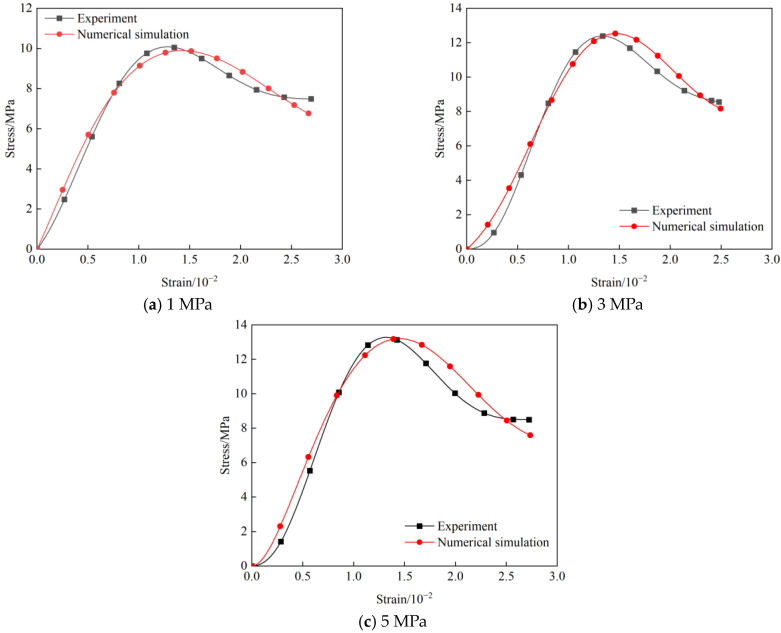
Stress–strain curves of different grouting pressure test rock samples and simulated rock samples.

To investigate the influence of grouting reinforcement on the mechanical behavior of rock samples, a comparative analysis of the stress–strain curves from experiments and numerical simulations was conducted for samples grouted at pressures of 1 MPa, 3 MPa, and 5 MPa, as shown in Figure 20. The results indicate that the simulated curves show a high degree of consistency with the experimental data in both overall trend and key characteristics, validating the effectiveness of the CT-reconstructed heterogeneous numerical model.

In the compaction stage, all grouted rock samples exhibited slight nonlinearity. The simulated curves closely matched the experimental curves in the initial portion and in the degree of nonlinearity, indicating that the model accurately characterized the initial state of the grouted rock samples.

During the elastic stage, the grouted rock samples displayed approximately linear behavior. A good agreement was observed between the simulated and experimental elastic moduli, with the simulated values being slightly lower. The smoothness of the simulation curve indicates that the definition of the contact surface and the setting of the boundary conditions are reasonable.

Upon entering the plastic stage, the simulations accurately captured the yield points and hardening behavior of each grouted rock sample, demonstrating that the *MAT_RHT model effectively describes composite plastic behavior. The plastic stage of the grouted rock samples was significantly prolonged compared to that of the ungrouted rock samples, illustrating the ductility enhancement due to grouting.

In the damage stage, the post-peak stress drop in grouted rock samples was more gradual than in ungrouted rock samples, indicating that grouting mitigated brittle characteristics. This feature was reproduced in the simulation through the coupling of *MAT_ADD_EROSION and the *MAT_RHT damage model, confirming the alteration of post-peak mechanical behavior by grouting reinforcement.

The comparative analysis reveals that the grouting pressure has a significant impact on the mechanical response. The simulations exhibited a high degree of agreement with the experiments in terms of elastic modulus, peak strength, and post-peak behavior, with deviations falling within an acceptable engineering range. Grouting not only enhances the strength of the rock samples but also modifies their deformation characteristics, particularly in the post-peak stage. Numerical simulation results fully validate the findings from laboratory testing.

**Figure 21:**
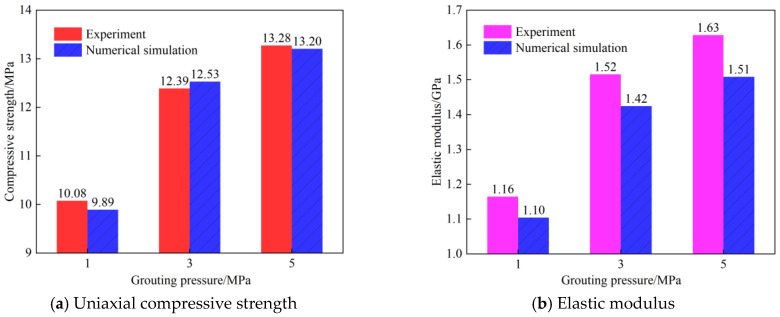
Compressive strength and elastic modulus of the test rock samples and simulated rock samples.

Figure 21 presents a comparative dual-axis bar chart of the experimental and simulated values for elastic modulus and compressive strength of rock samples grouted at 1, 3, and 5 MPa, enabling a quantitative analysis of the improvement in mechanical properties due to grouting.

The comparison of compressive strength reveals that grouting pressure significantly influences load-bearing capacity. For the sample grouted at 1 MPa, the experimental and simulated strengths were 10.08 MPa and 9.89 MPa, respectively, resulting in a deviation of 1.9%. The most substantial performance improvement was observed at 3 MPa grouting pressure, with experimental and simulated strengths reaching 12.39 MPa and 12.53 MPa (deviation 1.1%), representing a 26.7% increase over the 1 MPa condition. Further increasing the pressure to 5 MPa raised the strength to 13.28 MPa (experimental) and 13.20 MPa (simulated), with a deviation of 0.6%. However, this constituted only a 5.3% increase relative to the 3 MPa sample. The simulation accurately captured the trend of increasing strength with higher grouting pressure (5 MPa > 3 MPa > 1 MPa), validating the model’s effectiveness in reflecting the grouting enhancement effect.

The elastic modulus comparison highlights the improvement in stiffness due to grouting. The experimental moduli for the 1, 3, and 5 MPa grouted rock samples were 1.16 GPa, 1.52 GPa, and 1.63 GPa, respectively. The corresponding simulated values were 1.10 GPa, 1.42 GPa, and 1.51 GPa, yielding deviations of 5.2%, 6.6%, and 7.4%. The modulus increased by 29.1% under 3 MPa pressure, while the gain from 3 MPa to 5 MPa was only 6.3%. Although deviations exist, the simulated trend of increasing modulus aligns with the experimental data and remains within acceptable engineering tolerances. The pattern of modulus enhancement correlates with the strength improvement, further confirming the influence of grouting pressure on mechanical performance.

A comprehensive analysis indicates that the reinforcement effect of grouting improves nonlinearly with increasing pressure. Grouting at 3 MPa yielded the most significant leap in both strength and elastic modulus, representing the optimal improvement efficiency. While grouting at 5 MPa can further elevate the absolute values of these metrics, the marginal gain diminishes noticeably. The numerical simulation successfully reproduced the mechanical responses under different pressures, with deviations in compressive strength less than 2% and in elastic modulus less than 8%. This demonstrates the model’s reliable predictive capability and its suitability for engineering applications.

#### 5.2.2. Analysis of the Fracture and Failure Process in Grouted Rock Samples

**Figure 22:**
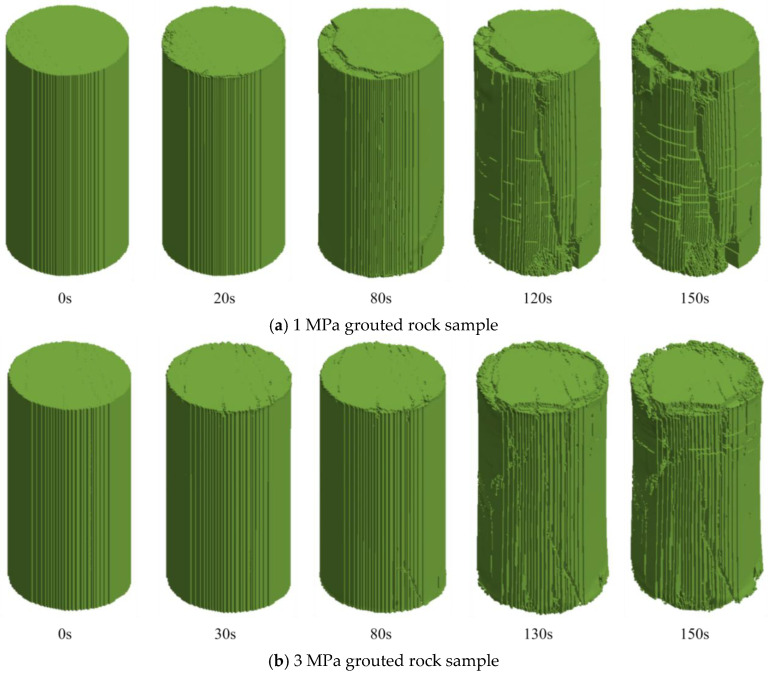

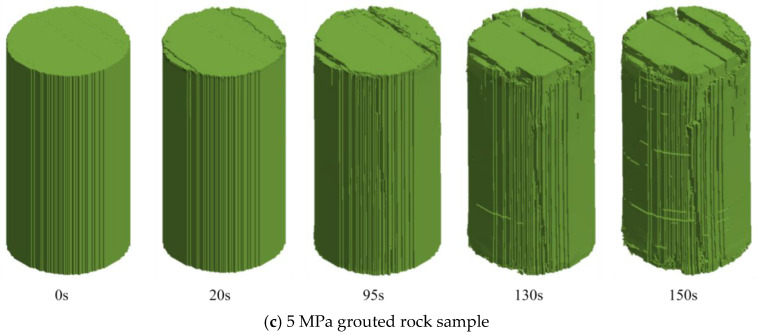
Failure process of grouted rock samples.

A systematic analysis of the simulation results from LS-DYNA was conducted to reconstruct the complete failure process of different grouted rock samples under uniaxial compression. Figure 22 illustrates the evolution of their failure morphology at different time steps.

In the initial stage, all rock samples maintained structural integrity. Stress was transmitted uniformly through both the rock skeleton and the grout matrix. The simulation showed good agreement with the initial compaction phase observed in the experiments.

Upon entering the elastic stage, initial damage began to develop. For the 1 MPa grouted rock sample, micro-cracks propagating along material interfaces appeared in grout-concentrated zones by 20 s. In contrast, only a few cracks were observed in the 3 MPa sample until 30 s. Although micro-cracks initiated in the 5 MPa sample by 20 s, they were scattered and the overall strength remained high. During this stage, cracks propagated stably, and the load-bearing capacity of the samples continued to increase, indicating that grouting delayed the onset of damage.

During the plastic development stage, damage accumulation accelerated. For the 1 MPa sample, micro-cracks coalesced between 20 and 80 s to form a dominant crack band. The 3 MPa sample exhibited stable damage progression between 30 and 80 s, with cracks displaying a dispersed distribution. In the 5 MPa sample, cracks propagated rapidly between 20 and 95 s. Overall, damage development in the grouted rock samples was slower and more stable, demonstrating enhanced ductility.

In the damage accumulation stage, macroscopic cracks began to form. The 1 MPa sample developed a thin, non-through-going primary crack by 80 s, coinciding with the onset of a decline in load-bearing capacity. The 3 MPa sample developed a relatively narrow, shear-oriented primary crack around the same time. For the 5 MPa sample, multiple cracks appeared by 95 s, which eventually evolved into a dominant failure surface.

In the final failure stage, the primary crack in the 1 MPa sample fully penetrated at 120 s, splitting the sample into two major blocks. The 3 MPa sample failed at 130 s, exhibiting a rough and tortuous failure surface. Failure in the 5 MPa sample also occurred at 130 s, but it retained a higher residual strength due to the reinforcement effect of the grout. All samples reached complete failure by 150 s, yet their failure modes differed significantly.

The comparison reveals that the grouting pressure has a decisive influence on the failure mode. The grouting process shifts the rock failure mechanism from sudden brittleness to progressive ductility, a finding of significant importance for engineering safety.

#### 5.2.3. Crack Evolution in Grouted Rock Samples

**Figure 23:**
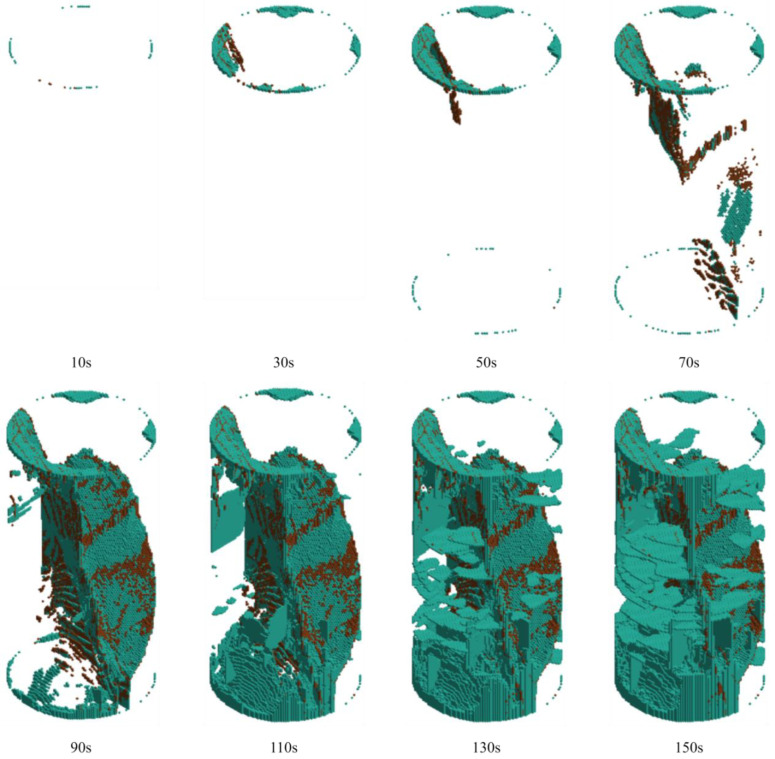
Crack evolution in 1 MPa grouted rock sample.

**Figure 24:**
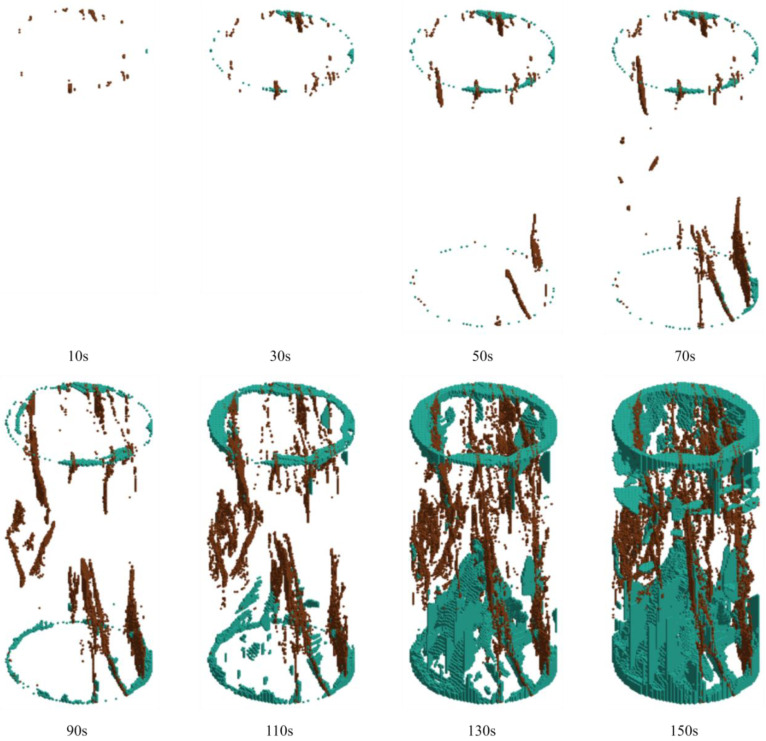
Crack evolution in 3 MPa grouted rock sample.

**Figure 25:**
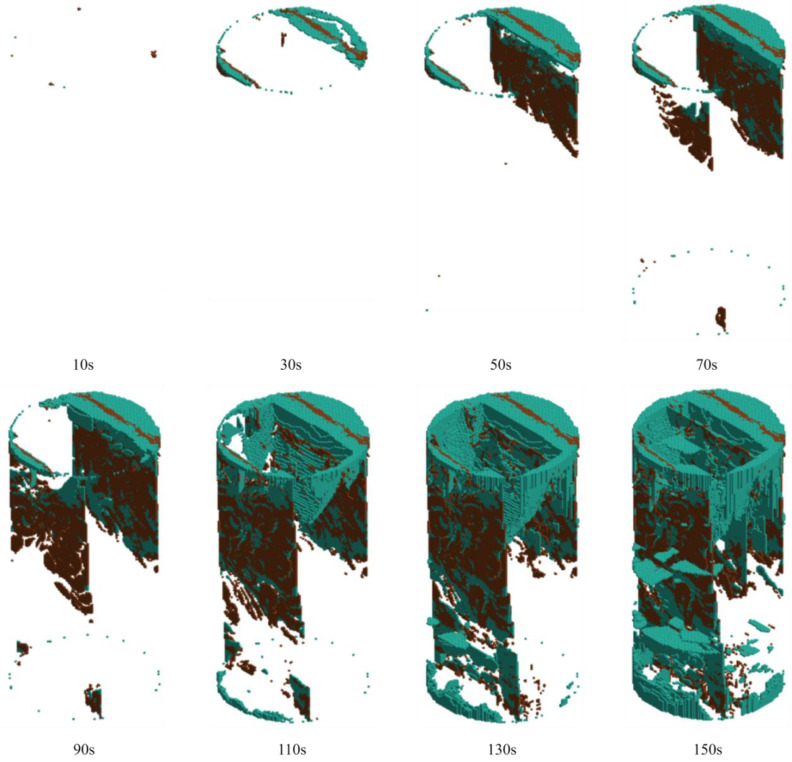
Crack evolution in 5 MPa grouted rock sample.

By tracking the failure units of LS-DYNA, the crack evolution laws of rock samples under different grouting pressures were systematically analyzed. The crack propagation morphologies of the grouted rock samples at various loading times are presented in Figure 23, Figure 24 and Figure 25. In these figures, brown cracks represent those propagating along pre-existing weak planes or the grout–rock interface, while cyan cracks indicate newly generated cracks during the loading process.

As shown in Figure 23, during the initial loading stage (10 s), cyan cracks first initiated in the stress concentration zones at the upper and lower ends of the specimen, appearing as fine radial cracks. No significant propagation of brown cracks was observed at this point. By 30 s, brown cracks began to extend into the interior of the sample, displaying a clear path preference. Concurrently, cyan cracks gradually developed in the end regions and started to connect preliminarily with the brown cracks. At 50 s, the brown cracks formed a distinct embryonic main crack, with a few new cyan cracks initiating around it. By 70 s, the width of the brown cracks showed negligible increase, and the propagation of cyan cracks remained relatively slow. After 90 s, the sample entered an accelerated failure stage. The propagation velocity of brown cracks increased sharply, and a large number of cyan cracks initiated and expanded rapidly. By 110 s, the dominant brown crack completely penetrated the specimen, forming a continuous failure surface, while cyan cracks densely developed around this main crack. At 130 s, obvious branching occurred in the cyan cracks, generating multiple secondary cracks on both sides of the main brown crack, with the two types of cracks developing in an intertwined manner. By 150 s, the final macroscopic failure surface was formed. This surface was dominated by the penetrated brown main crack, surrounded by a multi-crack interwoven zone consisting of cyan cracks, resulting in a relatively flat overall failure profile.

Throughout the entire failure process, the propagation rates of both crack types exhibited marked acceleration and abrupt changes. The brown cracks demonstrated a strong path dependency, whereas the cyan cracks displayed characteristics of sudden, explosive propagation. This indicates that grouting at 1 MPa provided limited reinforcement for the pre-existing weak planes, and the failure process was dominated by the rapid, synergistic propagation of both crack types, leading to complete structural failure.

The rock sample grouted at 3 MPa exhibited a progressive crack development pattern, as shown in Figure 24. At 10 s, only a small number of newly initiated cyan microcracks appeared locally in the central region of the specimen, distributed in a scattered, point-like manner. Brown cracks along pre-existing weak planes showed no significant development. By 30 s, both types of cracks began to propagate in an orderly fashion: brown cracks extended independently in multiple zones along weak planes, while cyan cracks were distributed more evenly, forming several independent crack nuclei, collectively resulting in a dispersed crack cluster. At 50 s, cyan cracks coalesced into distinct, fine parallel meso-cracks, while brown cracks extended correspondingly along weak planes. These cracks interacted and constrained each other, forming a stable and diversified crack network. By 70 s, an initial crack network had developed, with brown and cyan cracks interweaving without a clearly dominant main crack. After 90 s, the sample entered a stable failure phase, with the propagation rate of both crack types accelerating. At 110 s, multiple cyan and brown cracks began to converge toward the central region of the specimen, forming a relatively complex interwoven crack network. By 130 s, a primary failure surface had formed, with brown cracks showing a tendency to penetrate along weak planes, surrounded by densely developed cyan cracks, yet the specimen maintained good overall integrity. At 150 s, final failure occurred, with both crack types together constituting a rough yet continuous failure surface, and the rock sample retained residual strength. This indicates that 3 MPa grouting effectively coordinates the propagation of both crack types, enhancing the overall integrity and ductility of the rock sample.

The rock sample grouted at 5 MPa displayed stable failure characteristics, as illustrated in Figure 25. At 10 s, the specimen was in the initial stage, with only a few brown microcracks appearing in the upper part. By 30 s, both crack types initiated simultaneously at multiple locations in the specimen, with an initial number of 2–3 brown and cyan cracks each, and a distribution density noticeably lower than that in the other two sample groups. At 50 s, both crack types propagated rapidly and interconnected, forming a distinct crack network. Brown cracks assumed a dominant role earlier, showing accelerated propagation and a rapid increase in width, forming a main crack. By 70 s, the main brown crack had essentially penetrated the specimen, with cyan cracks extending synchronously around it. After 90 s, the sample entered an accelerated failure stage, with the propagation speed of both crack types further increasing. At 110 s, the width of the main brown crack continued to increase, accompanied by significant branching of cyan cracks. By 130 s, although a large number of secondary cracks did not appear, the density of the crack network increased significantly, with brown and cyan cracks developing in an intertwined manner, while the overall structure of the specimen did not disintegrate. Final failure occurred only at 150 s. The failure surface consisted of rough brown cracks along weak planes and ductile cyan new cracks, exhibiting clear ductile failure characteristics, and the rock sample retained residual strength. This indicates that 5 MPa grouting significantly enhances the ductility of the material and the controllability of the failure process.

Grouting pressure critically influences failure modes: The reinforcement effect of 1 MPa grouted rock samples is limited; 3 MPa provides effective crack suppression and structural integrity; and 5 MPa enhances strength and ductility but with diminishing returns. The 3 MPa condition demonstrates optimal crack control efficiency, maintaining high integrity and residual strength at relatively lower energy consumption.

#### 5.2.4. Analysis of the Equivalent Stress Cloud Map for Fracturing of Grouting Rock Samples

**Figure 26:**
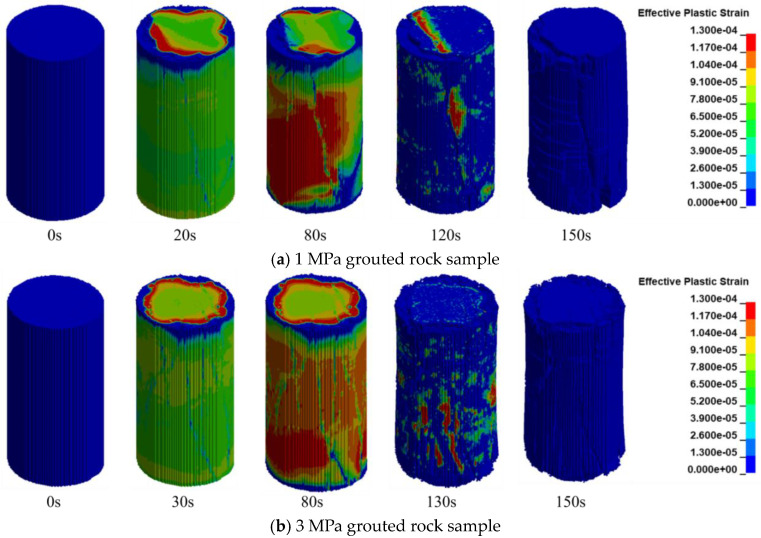

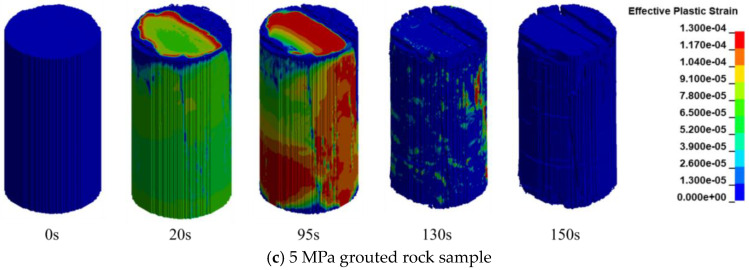
Equivalent stress contour plots of grouted rock samples.

The evolution of stress distribution in rock samples under different grouting pressures during uniaxial compression was systematically analyzed based on the equivalent stress contour plots output from LS-DYNA. Figure 26 presents the equivalent stress distribution (scale unit: 10^5^ MPa) for different grouted rock samples at various loading times.

Initial Loading Stage: Stress distribution was uniform in all samples. For the 1 MPa grouted rock sample, stress concentrations (2–4 MPa) appeared in grout-deficient zones between 0 and 20 s, coinciding with subsequent crack initiation locations. The 3 MPa sample exhibited slight stress concentrations at similar times, while the 5 MPa sample showed early stress gradients at the boundaries of grout-rich zones.

Elastic Development Stage: Stress concentrations became more pronounced. In the 1 MPa sample at 20 s, the concentrated zones expanded into bands, with the maximum stress reaching values represented by the red contour, predominantly located at discontinuous grout interfaces. The 3 MPa sample developed multiple dispersed stress concentration bands by 30 s, also reaching the red stress level. For the 5 MPa sample at 20 s, a stress peak of approximately 13 MPa occurred within grout-rich zones, which became the core areas for subsequent damage initiation.

Plastic Deformation Stage: Stress distribution became significantly differentiated. By 80 s, the 1 MPa sample formed interconnected high-stress bands aligning with the eventual main crack path. The 3 MPa sample maintained a relatively uniform stress distribution without forming a through-going high-stress band. In the 5 MPa sample at 95 s, multiple isolated high-stress zones appeared, mostly at the boundaries of grout matrix agglomerations, demonstrating an interfacial stress concentration effect. High-stress zones in all samples showed a development trend from the surface inward.

Damage Accumulation Stage: Stress distribution underwent drastic changes. In the 1 MPa sample at 120 s, stress in the original high-stress zones began to unload and transfer to adjacent areas, indicating active damage progression. The 3 MPa sample still maintained a relatively uniform, low-stress distribution at 130 s. For the 5 MPa sample at 130 s, high-stress zones decayed rapidly, and multiple stress-relieved areas connected to form macroscopic stress-release bands.

Final Failure Stage: By 150 s, the stress contour plots of all samples returned entirely to blue, indicating a complete loss of load-bearing capacity.

Comprehensive analysis indicates that grouting pressure significantly influences internal stress distribution. The 5 MPa condition resulted in the most pronounced stress concentration, while the 3 MPa condition exhibited a more uniform stress distribution and higher stress transfer efficiency. The grout–rock interface served as the primary stress concentration zone, with its stress gradient reflecting the quality of interfacial bonding. Stress evolution was highly coupled with damage development, and stress redistribution acted as the direct mechanism triggering and driving damage progression. The 3 MPa grouting condition achieved a favorable balance in stress homogenization and concentration control, contributing to the enhancement of the overall mechanical performance and failure toughness of the rock samples.

## 6. Discussion

This study systematically investigated the reinforcement effects of different grouting pressures on fractured sandy mudstone and the associated meso-mechanical mechanisms through integrated laboratory grouting tests, mechanical testing, SEM analysis, and CT-based in situ modeling coupled with numerical simulation. This section aims to provide a comprehensive assessment of the methodological advantages, mechanistic correlations, and engineering implications of the research while critically examining the limitations of the current work and proposing directions for future improvement.

The primary methodological innovation of this study lies in the development and application of a CT-based in situ modeling framework. Unlike conventional homogenized or idealized fracture simulation approaches, this study achieved high-fidelity reconstruction of the actual geometric configuration of the grout–rock composite by leveraging authentic CT scan data through image segmentation and mesh mapping techniques, thereby substantially enhancing both the geometric realism and meso-scale representational capability of the numerical model. SEM observations provided microstructural evidence supporting model parameterization: for instance, the sufficient filling of micropores by grout at 3 MPa corresponded to a marked increase in strength and stiffness within the model, whereas microcracks observed in the rock matrix adjacent to the interface under 5 MPa corroborated the phenomenon of excessive pressure inducing structural damage to the rock specimen itself. The validity of the model has been experimentally verified at two levels—macroscopic mechanical response and failure morphology.

Regarding numerical simulation, although the CT-based in situ modeling approach significantly improved simulation fidelity, certain limitations remain that warrant attention in both result interpretation and future research. The RHT constitutive model (*MAT_272) employed herein simplifies both rock and grout as ideal elastic–plastic materials, neglecting volume shrinkage during grout hardening, creep effects, and long-term chemical interactions with the surrounding rock, which may lead to an overestimation of long-term service performance. Furthermore, a direct mapping relationship between damage evolution in the model and actual physical damage mechanisms is lacking. Currently, model validation relies primarily on comparisons of macroscopic stress–strain curves and final failure patterns. Future work should integrate high-resolution full-field strain measurement techniques such as digital image correlation to achieve refined validation across the entire process—from localized strain concentration to global instability.

In terms of engineering implications, this study revealed a nonlinear threshold effect between grouting pressure and reinforcement efficacy in grouted rock specimens—identifying 3 MPa as the pressure level at which the marginal benefit of enhancement is maximized. However, direct translation of this pressure value to field-scale applications requires careful consideration of the complex characteristics of in situ fracture networks, including multi-branching, variable aperture, and the presence of infill materials. Grouting pressure undergoes significant attenuation along fracture pathways due to friction, filtration, and divergent flow. Moreover, field rock masses are subjected to three-dimensional in situ stress fields, and fracture dimensions far exceed those of laboratory-scale specimens. The optimal grouting pressure may vary with reinforcement depth, in situ stress orientation, and fracture geometry. Future research should integrate field grouting trials with numerical models incorporating complex fracture networks to establish quantitative correlations between laboratory-derived threshold pressures and field construction parameters.

In conclusion, this study has established a preliminary bridge between method innovation, mechanism revelation and practical engineering, but it still needs to be continuously deepened in aspects such as model refinement, verification diversification and application scenario expansion.

## 7. Conclusions

This study systematically revealed the reinforcement laws and damage mechanisms of grouting pressure on the mechanical properties of fractured sandy mudstone through an integrated approach combining laboratory tests, meso-scale characterization, and numerical simulation. A numerical simulation method for grout–rock composite structures based on CT in situ modeling was established. The main conclusions are as follows:

(1) The reinforcement effect of grouting pressure on fractured sandy mudstone exhibits a nonlinear enhancement pattern. When the grouting pressure was increased from 1 MPa to 3 MPa, the average peak strength and elastic modulus of the rock specimens increased significantly (by 22.9% and 31.0%, respectively). However, further increasing the pressure to 5 MPa resulted in a markedly diminished rate of improvement. A grouting pressure of 3 MPa was identified as optimal under the tested conditions, achieving the best balance among strength gain, stiffness enhancement, and damage control.

(2) Grouting transformed the failure mode of the rock specimens from brittle-dominated to ductile-dominated behavior. As the grouting pressure increased, the average toughness index continuously increased (1.6555 → 1.7135 → 1.7648), the brittleness index progressively decreased (1.5255 → 1.4020 → 1.3075), and the ductility index exhibited a marked increase (1.8760 → 2.0972 → 2.2637).

(3) SEM analysis revealed that under a grouting pressure of 1 MPa, failure was dominated by wide primary cracks. As the grouting pressure increased to 3 MPa, the grout fully penetrated into micropores and interparticle voids, significantly enhancing the overall integrity of the rock specimens. At 5 MPa, although the grout distribution further expanded, grouting-induced microcracks appeared within the rock matrix.

(4) This research established an integrated numerical simulation methodology combining “CT scanning—in situ modeling—mechanical analysis”. This approach successfully achieves authentic in situ modeling of the grout–rock composite structure. It provides a reliable method for the in-depth analysis of grouting reinforcement mechanisms and parameter optimization.

Practical implications for engineering design of grouting reinforcement in fractured rock masses: Grouting pressure is not necessarily better when higher; there exists an optimal interval from a mechanical perspective. Blind pursuit of high pressure not only yields diminishing returns but may also induce new damage to the rock mass, increasing engineering costs and safety risks. Furthermore, the integrated “CT scanning—in situ modeling—mechanical analysis” methodology established in this study provides a reliable approach for in-depth analysis of grouting reinforcement mechanisms and parameter optimization. Directions for future research: Based on the limitations of this study, future research will focus on the following directions: (1) Material dimension: Systematically evaluate the effects of different grout mix proportions (e.g., nanomaterial content, water-to-cement ratio) and admixture types on reinforcement performance, and establish a correlation model linking grout composition, meso-structure, and macroscopic properties. (2) Structural dimension: Consider the diversity of fracture occurrences (dip angle, aperture, roughness) and network topology (branching, intersection, density) to construct more universally applicable mechanical models for grouting reinforcement. (3) Temporal dimension: Conduct long-term durability tests under wet–dry cycles, chemical erosion, and creep loading to reveal the time-dependent degradation laws of grout–rock interfacial bonding performance.

## Figures and Tables

**Table 1:** Chemical composition of ordinary Portland cement, aluminate cement and fly ash.

Materials	CaO	Al_2_O_3_	SiO_2_	Fe_2_O_3_	MgO	Na_2_O	K_2_O	SO_3_	Others
OPC/%	51.42	8.26	24.99	4.03	3.71	0.18	0.63	2.21	4.57
CA/%	35.85	55.39	4.84	1.63	—	0.17	0.07	—	2.05
FA/%	5.61	29.24	50.21	3.65	1.62	2.14	1.31	2.11	4.11

**Table 2:** Mix ratio of grouting material.

Component	Cementitious Base			Additives				Water–Cement Ratio
	Portland Cement/%	Aluminate Cement/%	Fly Ash/%	Nano-Silica Sol/%	EVA/%	Water Reducer/%	Defoamer/%	
Content	80	5	15	2	7.5	1	0.3	0.7

**Table 3:** Physical and mechanical parameters of grouting material.

Grouting Material Properties			Material Properties of 28d Stone Body		
Fluidity /mm	Initial Setting Time/min	Hardening Rate/%	Compressive Strength/MPa	Tensile Strength/MPa	Compressive-Tensile Ratio/%
300	165	97.6	16.63	2.82	0.17

**Table 4:** Toughness and brittleness indices of grouted rock samples.

Grouting Pressure	1 MPa	Average Value	3 MPa	Average Value	5 MPa	Average Value
Toughness index	1.6289	1.6555	1.6958	1.7135	1.7415	1.7648
	1.6572		1.7142		1.7823	
	1.6804		1.7305		1.7814	
Brittleness index	1.5082	1.5255	1.3568	1.4020	1.2856	1.3075
	1.5319		1.4192		1.3189	
	1.5364		1.4300		1.3180	

**Table 5:** Ductility index of grouted rock samples.

Grouting Pressure	1 MPa	Average Value	3 MPa	Average Value	5 MPa	Average Value
*μ*	1.8327	1.8760	2.0285	2.0972	2.1852	2.2637
	1.8854		2.1063		2.2705	
	1.9099		2.1568		2.3354	

**Table 6:** RHT model parameter values.

Parameter	Grout Matrix	Rock Matrix	Parameter	Grout Matrix	Rock Matrix
$\rho_{0}\left(kg/m^{3}\right)$	2380	2610	$\beta_{t}$	0.091	0.011
$G\left(GPa\right)$	1.12	6.35	PFC	0.001	0.001
EPSF	2	2	$g_{c}^{*}$	0.53	0.53
$B_{0}$	1.22	1.22	$g_{t}^{*}$	0.7	0.7
$B_{1}$	1.22	1.22	XI	0.5	0.5
$T_{1}\left(GPa\right)$	4.12	35.27	$D_{1}$	0.04	0.04
A	2.643	1.60	$D_{2}$	1	1
N	0.668	0.61	$\varepsilon_{p}^{m}$	0.02	0.01
$f_{c}\left(MPa\right)$	12.7	67.8	$A_{f}$	1.78	1.6
$f_{s}^{*}$	0.18	0.18	$n_{f}$	0.8	0.61
$f_{t}^{*}$	0.202	0.04	GAMMA	0	0
$Q_{0}$	0.68	0.68	$A_{1}\left(GPa\right)$	4.12	35.27
B	0.05	0.01	$A_{2}\left(GPa\right)$	5.03	39.58
$T_{2}$	0	0	$A_{3}\left(GPa\right)$	1.06	9.04
${\dot{\varepsilon }}_{0}^{c}$	3 × 10^−5^	3 × 10^−5^	$p_{el}\left(MPa\right)$	13.3	125
${\dot{\varepsilon }}_{0}^{t}$	3 × 10^−6^	3 × 10^−6^	$p_{comp}\left(GPa\right)$	6	6
${\dot{\varepsilon }}^{c}$	3 × 10^25^	3 × 10^25^	$n_{p}$	3	3
${\dot{\varepsilon }}^{t}$	3 × 10^25^	3 × 10^25^	$\alpha_{0}$	0	0
$\beta_{c}^{t}$	0.154	0.0076			

**Table 7:** RHT parameter values under different grouting pressures.

Parameter	1 MPa Grouting Pressure	3 MPa Grouting Pressure	5 MPa Grouting Pressure
$f_{c}\left(MPa\right)$	14.15	15.24	16.33
$f_{t}^{*}$	0.218	0.230	0.240
A	2.7	2.82	2.95
N	0.67	0.68	0.69
$A_{1}\left(GPa\right)$	4.3	4.65	5.00
$A_{2}\left(GPa\right)$	5.25	5.68	6.10
$A_{3}\left(GPa\right)$	1.15	1.30	1.45
$p_{el}\left(MPa\right)$	14.5	16.5	18.5
$\varepsilon_{p}^{m}$	0.021	0.023	0.025

## Data Availability

Data will be made available on request. All relevant data are available upon reasonable request. For data requests, please contact corresponding author Zhaoyun Chai (chaizhaoyun@tyut.edu.cn).
